# Prevalence, progression, and clinical outcomes of mitral valve prolapse: a systematic review and meta-analysis

**DOI:** 10.1093/ehjqcco/qcaf016

**Published:** 2025-03-28

**Authors:** Thalia Melamed, Sveeta Badiani, Stephen Harlow, Nabila Laskar, Thomas A Treibel, Nay Aung, Sanjeev Bhattacharyya, Guy Lloyd

**Affiliations:** Faculty of Medicine and Dentistry, Queen Mary University of London, Garrod Building, Turner St, London E1 2AD, UK; Barts Heart Centre, St Bartholomew's Hospital, London EC1A 7BE, UK; Faculty of Medicine and Dentistry, Queen Mary University of London, Garrod Building, Turner St, London E1 2AD, UK; Barts Heart Centre, St Bartholomew's Hospital, London EC1A 7BE, UK; Barts Heart Centre, St Bartholomew's Hospital, London EC1A 7BE, UK; Barts Heart Centre, St Bartholomew's Hospital, London EC1A 7BE, UK; Barts Heart Centre, St Bartholomew's Hospital, London EC1A 7BE, UK; Barts Heart Centre, St Bartholomew's Hospital, London EC1A 7BE, UK

**Keywords:** Mitral valve prolapse, Prevalence, Epidemiology, Meta-analysis, Mitral regurgitation, Outcomes

## Abstract

**Aims:**

The prevalence of mitral valve prolapse (MVP) varies across populations and age groups; its natural history and clinical outcomes remain unclear. This meta-analysis established the prevalence of MVP in the general population, in associated syndromes and at different ages. It also determined the rate of progression and the incidence of adverse outcomes.

**Methods and results:**

A systematic search identified original reports on the prevalence of MVP and related outcomes. A total of 83 studies met inclusion: 47 (*n* = 992 944) non-syndrome associated; 31 (*n* = 3067) syndrome associated; and 5 (*n* = 1287) described mitral regurgitation (MR) progression or adverse outcomes. In the general population, the prevalence was 1.35% but higher in hospital cohorts (8.7%). Age-stratified prevalence was 0.5, 1.8, 2.7, and 2.0% in neonates, children, adolescents and adults, respectively. Meta-regression and subgroup analysis found no significant difference (*P* = 0.81) across ages but revealed a significantly higher prevalence in older compared to young adults (2.87% vs. 0.67%, *P* = 0.01). Prevalence rates were markedly higher in patients with genetic syndromes. MR progressed at 5.5 per 100 person-years, overall. Event rates for all-cause mortality, development of heart failure, and need for mitral valve intervention were 1.7, 1.0, and 1.2 per 100 person-years, respectively.

**Conclusion:**

MVP is common, with greater prevalence in syndromes. Although more common with age, MVP is observed in infants. MVP related MR is progressive, especially in moderate MR, and there is a signal of excess mortality for unclear reasons. Valve services must manage the whole life journey and the potential risks associated with MVP.

## Graphical Abstract

**Graphical Abstract:**
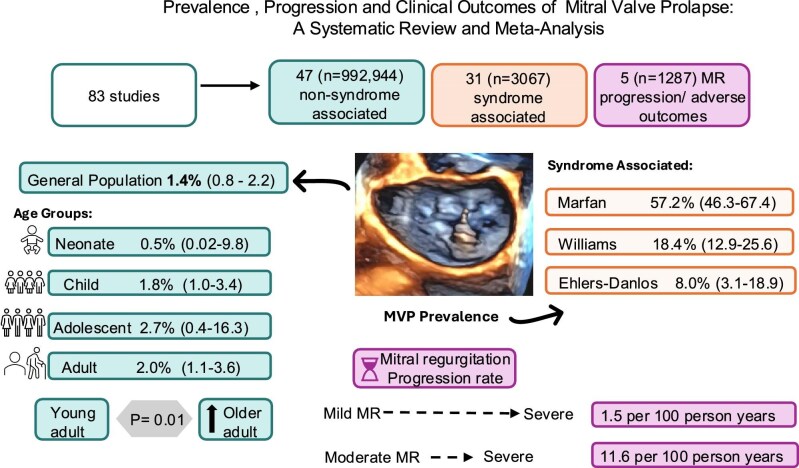

Key Learning Points **What is already known**

- The prevalence of mitral valve prolapse (MVP) is lower than previously described as earlier diagnostic methods were less specific, resulting in elevated false-positive rates.
- MVP is a known cause of mitral regurgitation, but the rate of disease progression is incompletely understood.

**What this study adds**

- This study estimates a MVP prevalence of 2.6%, with a general population prevalence of 1.4% and significantly higher in hospital cohorts (8.7%), showing notable variability across sub-populations and highlighting potential high-risk groups.
- This meta-analysis shows MVP is observed in infancy, increases with age, and is most prevalent among older adults (2.9%).
- This study demonstrates both a substantial progression rate especially for those with moderate mitral regurgitation and a concerning signal of excess mortality for which the cause is unclear, as patients with known or suspected arrhythmic MVP at source were excluded.
- Our findings highlight the need for valve services to manage the whole life journey and the potential risk associated with MVP.

## Introduction

Mitral valve prolapse (MVP) refers to the superior displacement of one or both mitral valve leaflets into the left atrium (LA) during ventricular systole.^1^

Historically, estimates of MVP prevalence have varied widely, reaching as high as 35%, partly because of early reliance on less specific diagnostic methods.^2,3^ Over time, MVP diagnostic criteria on echocardiography have evolved with a better understanding of the mitral valve anatomy, reducing perceived overdiagnosis.^4^ This has been suggested in a recent systematic review by Sonaglioni et al.,^5^ but there are no meta-analyses reporting the dimension of this problem. Inconsistencies in reported prevalence persist; estimates range from 0.6 to 3.4% in the adult general population, with variations observed across different populations and age groups.^1,6–13^

The age at which MVP becomes apparent remains unclear and its initiation and progression across various life stages is inadequately defined. Delling et al., demonstrated that adults with non-diagnostic MVP morphologies on echocardiography may evolve to fully diagnostic MVP, indicating the possibility of an early MVP phenotype.^6^ However, these findings cannot be generalized to younger populations. It is uncertain whether findings in children and adolescents represent a true phenotypic expression or reflect overdiagnosis from methodological differences.

Aetiology is likewise poorly understood; it clearly has a genetic basis, being strongly associated with Marfan's syndrome and other connective tissue disorders, termed *syndromic* MVP.^2^ In patients with no known genetic disorder, there is some but relatively weak evidence of a familial relationship.^2,14^

MVP is the leading cause of mitral regurgitation (MR) requiring surgery, but its natural history and disease progression rate is incompletely understood.^15–19^ While MVP is generally associated with a benign prognosis, there is a subset of the population at risk of developing adverse clinical outcomes, including heart failure, need for mitral valve intervention, and sudden death.^19,20^

In view of these uncertainties, we undertook a comprehensive meta-analysis to better understand the prevalence of MVP across different populations, age groups, and diagnostic criteria, to determine the probability of MR progression and the incidence rate of clinical events.

## Methods

### Protocol registration

The systematic review and meta-analysis were registered in the International Prospective Register of Systematic Reviews database under protocol CRD42023451697. They were performed in accordance with the recommendations by the Meta-Analysis of Observational Studies in Epidemiology group and the Preferred Reporting Items for Systematic Reviews and Meta-analysis (PRISMA).^21,22^

### Search strategy

The main aims of this review were to assess:

- the prevalence of MVP in the general population and specific cohorts, including recognized associated syndromes,
- the probability of MR progression by at least one grade,
- the incidence rate of adverse clinical events in MVP, as defined by development of heart failure, need for mitral valve intervention and all-cause mortality.

A systematic search was conducted using MEDLINE, EMBASE, and The Cochrane Library from inception to July 2023. Each endpoint was addressed with a separate search and results were amalgamated to streamline the screening and selection of studies. The detailed search strategies are included in the supplementary document. A medical librarian was consulted to refine the strategy and identify key search terms.

Two authors (T.M. and S.B.) independently performed the screening of titles and abstracts, reviewed the full text articles and determined their eligibility according to predefined criteria. Should there be any disagreements not resolved by consensus, a third reviewer was consulted (G.L.).

## Eligibility criteria

### Prevalence

Inclusion criteria were: (i) cross sectional, retrospective or prospective observational studies; (ii) that reported MVP prevalence; and (iii) diagnosed by 2-dimensional transthoracic echocardiography. No restrictions were placed on the specific criteria used based on 2 key reasons. First, we sought to assess the prevalence of MVP with increasing age, including neonates and young children, and the diagnostic criteria proposed by Freed et al.^1^ may not apply uniformly. Second, to allow examination of MVP prevalence based on varying echocardiographic standards. Both single and bileaflet MVP were included regardless of leaflet thickness.

Exclusion criteria were: (i) MVP diagnosis based solely on auscultation, M-mode or phonocardiography; (ii) no mention of the mode of MVP diagnosis or if there was inconsistency amongst participants with some not based on 2DTTE; (iii) an exclusively arrhythmic phenotype cohort to avoid misrepresentation of prevalence estimates, as it may represent a distinct clinical subset;^23^ (iv) case reports, case series, conference abstracts, reviews, and (v) studies not published in English language. This ensured the ability of the authors to accurately interpret the methodologies and findings of the included studies and maintain the highest standards of rigor in the systematic review and meta-analysis.

### MR progression

Inclusion criteria were: (i) prospective or retrospective observational studies (ii) including patients with MVP; (iii) diagnosed by 2DTTE, with MVP defined by >2 mm displacement of one or both mitral leaflets into the LA in the long-axis view; (iv) with at least 1 year follow up time; and (v) reporting progression of MR severity.

We excluded studies (i) with populations restricted to severe MR; (ii) including an exclusively arrhythmic phenotype cohort; (iii) MVP diagnosis based solely on auscultation, M-mode or phonocardiography; (iv) unspecified diagnostic method or inconsistent amongst participants with some not based on 2DTTE; (v) case reports, case series, conference abstracts, and reviews, and (vi) studies not published in English language.

### Adverse clinical event

Inclusion criteria were: (i) prospective or retrospective observational studies (ii) including patients with MVP; (iii) diagnosed by 2DTTE with MVP defined by >2 mm displacement of one or both mitral leaflets into the LA in the long-axis view; (iv) with at least 1 year follow up time; (v) reporting need for mitral valve intervention, (vi) development of congestive heart failure, and (vii) mortality.

Exclusion criteria were: (i) studies comprising solely of an arrhythmic phenotype patient population because prognosis may be specific to this cohort, and analysis goes beyond the remit of this study; (ii) MVP diagnosis based solely on auscultation, M-mode or phonocardiography; (iii) unspecified or inconsistent MVP diagnostic methods not based on 2DTTE; (iv) case reports, case series, conference abstracts, and reviews, and (v) studies not published in English language.

Uniform diagnostic criteria were required for studies reporting MR progression or adverse outcomes in MVP to ensure consistency and to avoid misrepresentation of these outcomes.

### Data extraction

Data were extracted by (T.M.) and (S.B.). Study characteristics: first author, year of publication, country, study design, criteria for MVP diagnosis, and population characteristics: population studied, sample size, *n* diagnosed with MVP, mean age, and sex (proportion of females screened) were collated for all eligible studies alongside the outcomes of interest (Supplementary material online, *Tables S1–S4*). Prolapse localization, baseline MR severity, MR diagnostic criteria, left ventricular ejection fraction (%), and follow up period (years) were further collected for studies reporting MR progression and adverse clinical events (Supplementary material online, *Table S5*).

### Subgroup analysis

The following subgroup analysis were conducted:

- By age categories (excluding syndrome patients), divided into neonates, children, adolescents and adults, defined by age ranges of <28 days, 1–11 years, 12–18 years, and >18 years. Children in the age range between 29 days and 364 days have not been included due to unavailability of data from the included studies.
- Young adults, with age range defined as 18–40 years vs. older subjects. This classification aligns with the age ranges of the included studies.
- Community and systematic screening studies vs. hospital cohort studies.
- Diagnostic criteria used for MVP diagnosis:
  - Displacement of one or both mitral leaflets into the LA in any view.
  - Displacement of one or both mitral leaflets into the LA in the parasternal long axis view (PLAX).
  - ≥2 mm displacement of one or both mitral leaflets into the LA in any view.
  - ≥2 mm displacement of one or both mitral leaflets into the LA in the PLAX view.

When considering the probability of MR progression, sub analyses were conducted according to baseline MR severity and study design (prospective or retrospective).

### Assessment of methodological quality and risk of bias

The quality of included studies was independently assessed by two reviewers (T.M. and S.H.). To assess the risk of bias for each paper, the Joanna Briggs Institute Critical Appraisal Checklist^24^ was used for prevalence studies, and the Quality in Prognosis Studies tool^25^ for studies reporting MR progression and related adverse clinical events.

### Data synthesis

To address study differences in follow up duration for time related outcomes, total person follow-up years was calculated. A detailed explanation is provided in the Supplementary material online, Page 1.

### Statistical analysis

The meta-analysis was conducted using R statistical software version 4.2.3, using the function ‘metaprop()’ from the ‘meta’ package. The pooled estimates for both prevalence and event rates and their 95% confidence intervals (CIs) were calculated using Der Simonian Laird Random effects model. In preparing the data for pooling, 0.5 was added to event counts equal to zero to ensure numerical stability and prevent potential overestimated results. Variance stabilization was achieved through a LOGITS transformation of the data. The final pooled results and their 95% CIs were back transformed and expressed as a percentage for interpretability. The Inverse variance method was used for weighing studies within the meta-analysis.

Publication bias was assessed by funnel plot symmetry. Egger's regression test was conducted when plot appeared asymmetrical on visual inspection.

### Handling heterogeneity

Heterogeneity was assessed using the *I* squared statistic (*I*^2^) which indicates the variability in pooled results across studies beyond what would be expected due to chance alone. *I*^2^ indexes of <25, 25–50, and >50% were considered low, medium, and high levels of heterogeneity, respectively.

To investigate potential sources of heterogeneity, subgroup, previously described, as well as sensitivity analyses were performed by restricting analysis to broad community-based studies, studies with a low risk of bias and leave-one-out analysis to evaluate single study impact. We also assessed the consistency of results across diverse analytical approaches (Supplementary material online, *Page 15*).

### Meta regression

Univariate meta-regression was conducted to explore potential associations between MVP's prevalence with age (mean age) and gender (proportion of females).

Statistical significance for pooled estimates was set at *P* < 0.05 (two-tailed).

## Results

### Study selection and patient population

**Figure 1:**
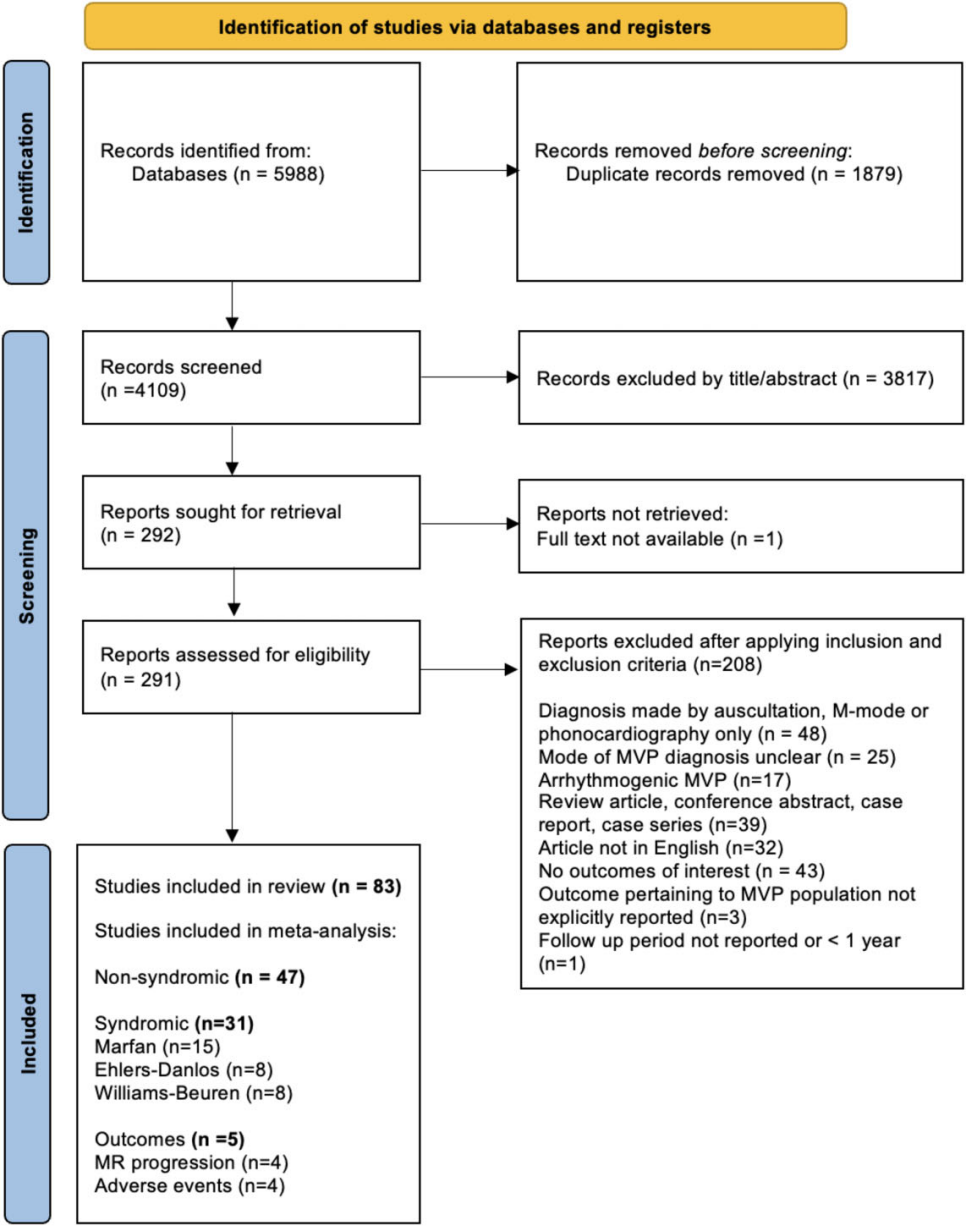
PRISMA flow diagram for study screening and selection. A total of 83 studies were included: 47 non-syndrome associated MVP, 31 syndrome associated, and 5 studies reporting progression or outcomes.

A total of 5988 publications were identified. Of these, 292 studies were selected for full text review, with 83 included in this analysis after pre-specified exclusions (*Figure 1*). Forty seven studies (*n* = 992 944) documented the prevalence of MVP in non-syndrome associated populations and 31 studies (*n* = 3067) focused on known associated syndromes, of which 15, 8, and 8 related to Marfan's, Ehlers–Danlos, and Williams syndromes, respectively. Four articles documented the rate of MR progression in 454 patients with MVP during a mean follow up period of 7.8 ± 3.7 years. Four studies reported the incidence of adverse clinical events. Population and study characteristics of the included studies are presented in the Supplementary material online, *Tables S1*–S5 for general population, syndromes, and outcome studies, respectively.

### Overall prevalence

**Figure 2:**
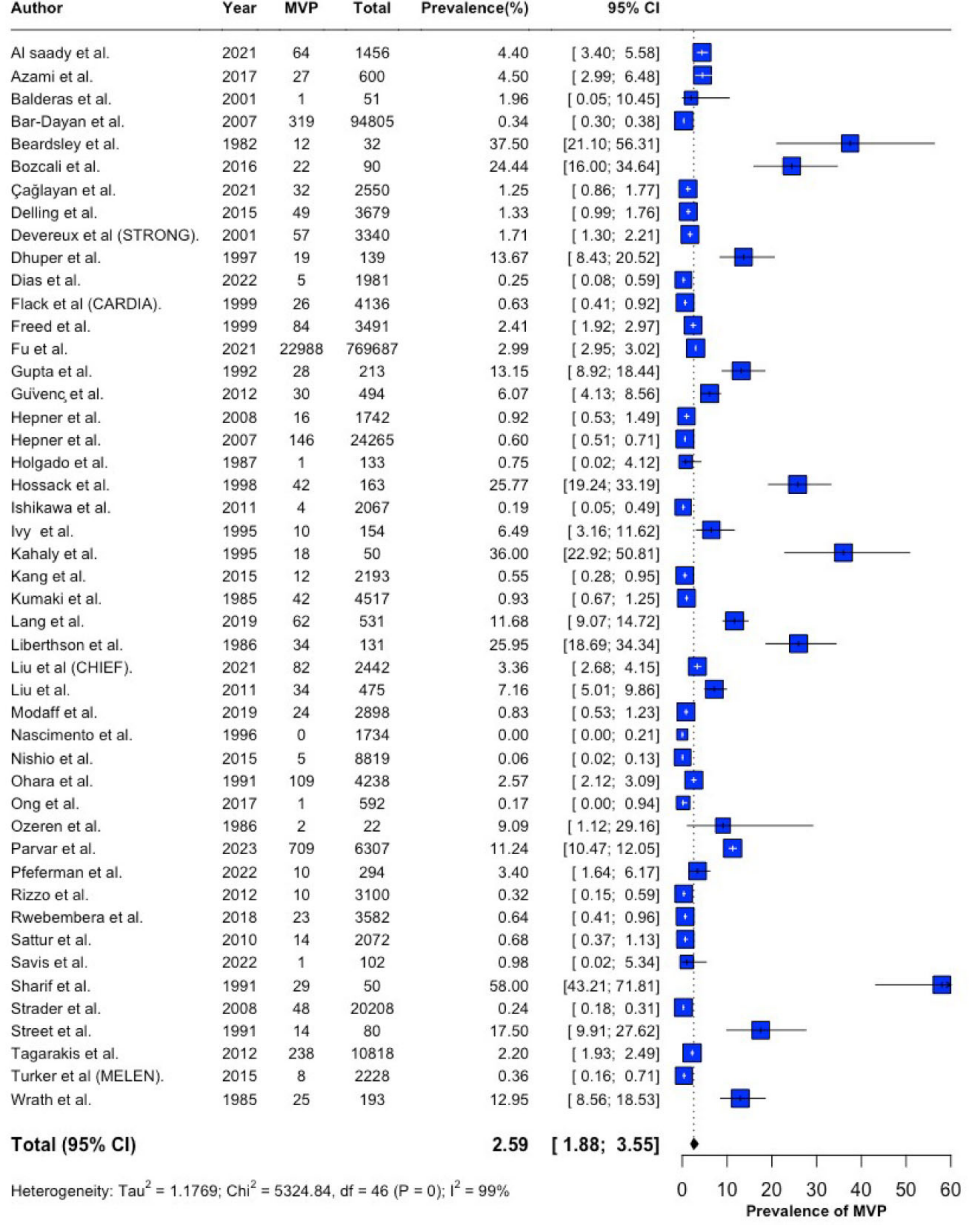
Forest plot showing the overall prevalence of MVP.

**Figure 3:**
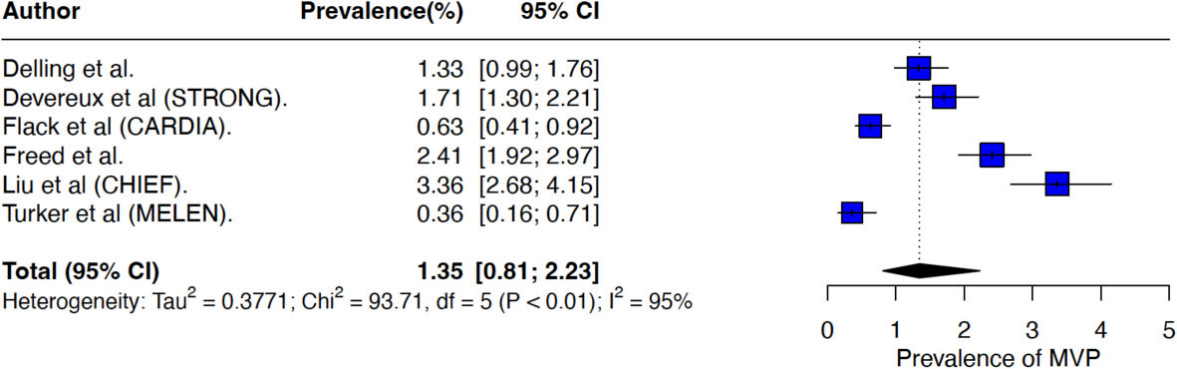
Forest plot showing the pooled prevalence of MVP in the general population.

The overall pooled prevalence of MVP was 2.59% (95% CI 1.88–3.55%, *I*^2^ = 99%) (*Figure 2*), with significant heterogeneity across studies. Leave-one-out analysis indicated that no single study demonstrated a disproportionate influence on the pooled prevalence (Supplementary material online, *Figure S1*). Application of alternative analytical methods outlined in the Supplementary material online, *Table S6* produced similar estimates and did not reduce the observed heterogeneity. Sensitivity analysis of studies with low risk of bias remained consistent with the result of the main analysis (Supplementary material online, *Figure S2*) and a symmetrical funnel plot supported a low risk of publication bias (Supplementary material online, *Figure S3*). When the analysis was limited to community studies and systematic screening studies, a significantly lower MVP prevalence (0.9%) compared to hospital cohort studies (8.7%) was observed (Supplementary material online, *Figure S4*). Restricting meta-analysis to broad community studies of adults with strict diagnostic criteria (*n* = 6), resulted in a pooled prevalence of 1.35% (95% CI 0.81–2.23, *I*^2^ = 95%) (*Figure 3*). No gender-based prevalence differences were observed (Supplementary material online, *Figure S5*).

### Age groups

Subgroup analysis, stratifying MVP prevalence based on age groups found the prevalence in neonates, children, adolescents and adults to be 0.49, 1.81, 2.70, and 2.02%, respectively, although the observed differences were not statistically significant (*P* for subgroup difference = 0.81) (Supplementary material online, *Figure S6*). The adolescent group accounted for the highest observed prevalence. However, when the study by Warth et al. was removed from the pooled analysis, the prevalence in the adolescent group decreased to 1.06% (95% CI 0.15–7.05%, *I*^2^ = 99%). Further analysis highlighted a significantly greater prevalence in older adults compared to young adults (2.87% vs. 0.67%, *P* = 0.01, *I*^2^ = 98%) (Supplementary material online, *Figure S7*). Taken together, there seems to be an increasing MVP prevalence with age; however, the lack of statistical significance indicates the need for caution when interpreting these findings.

### Echocardiography diagnostic criteria

**Figure 4:**
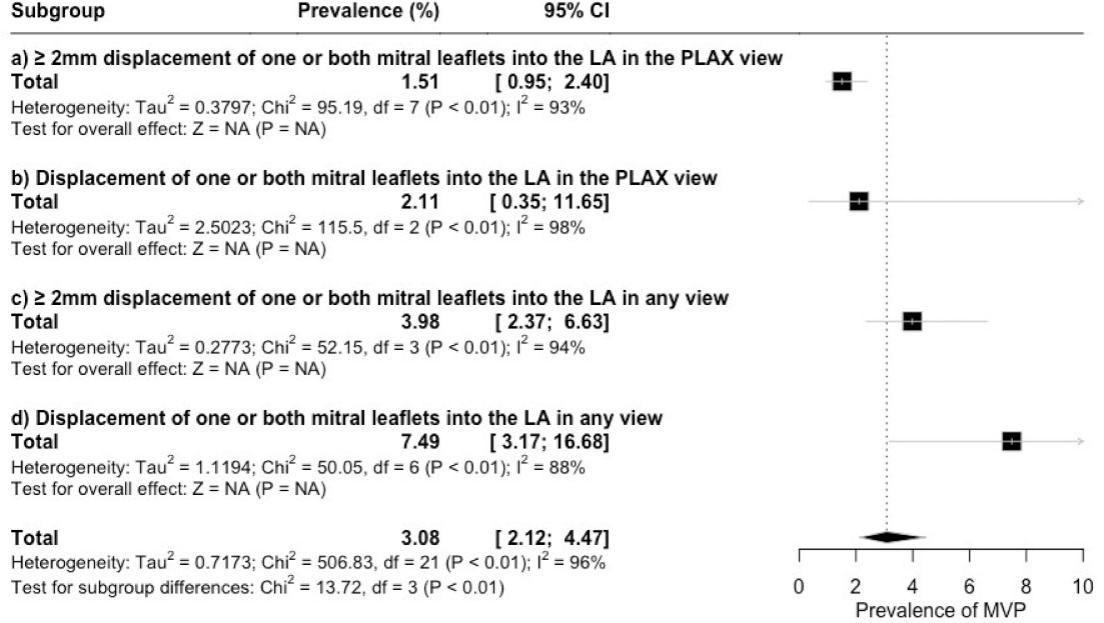
Forest plot displaying the stratified prevalence of MVP according to echocardiographic criteria.

Subgroup analysis based on echo diagnostic criteria demonstrated significant variations in prevalence estimates (*P* < 0.01) (*Figure 4* and Supplementary material online, *Figure S8*). The highest prevalence was observed when the diagnosis considered leaflet displacement visible in any echocardiographic view [7.49% (95% CI 3.17–16.68%, *I*^2^ = 98%)]. The lowest prevalence was found when MVP diagnosis was limited to >2 mm leaflet displacement in the PLAX view, at 1.51% (95% CI 0.95%–2.40%, *I*^2^ = 93%). When the criteria included any degree of displacement in the PLAX view and >2 mm displacement observed in any echo view, the pooled prevalence's were 2.11% (95% CI 0.35–11.65%, *I*^2^ = 98%), and 3.98% (95% CI 2.37–6.63%, *I*^2^ = 94%), respectively.

### Prevalence in syndromes

MVP affected patients with syndromes at much higher prevalence rates. The overall pooled estimate was 30.7% (95% CI 21.8–41.3%, *I*^2^ = 95%) (Supplementary material online, *Figure S9*). Marfan's syndrome was associated with the highest prevalence, at 57.2% (95% CI 46.3–67.4, *I*^2^ = 90%), followed by Williams at 18.4% (95% CI 12.9–25.5%, *I*^2^ = 73%) and Ehlers–Danlos at 8% (95% CI 3.1–18.9%, *I*^2^ = 94%). Between study heterogeneity was high (*I*^2^ = 95%), which was not accounted for by statistical sources of heterogeneity (Supplementary material online, *Table S6*). The study by Shiari et al., showed a disproportionate prevalence of MVP in Ehlers–Danlos, possibly due to its small sample size and inclusion of individuals referred to a tertiary-referral centre, with the possibility of overdiagnosis in this selected cohort. Leave-one-out analysis did not show any study particularly influenced the pooled estimate (Supplementary material online, *Figure S10*). Funnel plot appeared symmetrical (Supplementary material online, *Figure S11*), supporting a low risk of publication bias.

### MR progression

**Figure 5:**
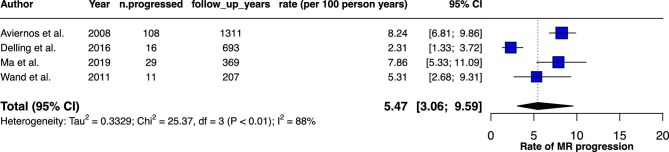
Forest plot illustrating the rate of MR progression by at least one grade associated with MVP.

Four studies assessed MR progression. Overall, the probability of MR progression by at least one grade was estimated to be 5.5 per 100 person-years (*Figure 5*). Subgroup analysis showed that patients with moderate compared to mild MR at baseline exhibited significantly faster rates of progression towards severe MR (11.6 vs. 1.5 per 100 person-years, *P* = 0.02 (Supplementary material online, *Figure S12*). Notably, heterogeneity among studies decreased substantially, particularly in the moderate MR group, where the heterogeneity index dropped to 0%. There was no significant difference in the rate of progression in retrospective and prospective studies (Supplementary material online, *Figure S13*).

### Adverse clinical events

#### All-cause mortality

Three studies reported the incidence of all-cause mortality in 967 patients during a mean follow up period of ∼9*±* 8 years. The pooled incidence rate of all-cause mortality was 1.7 (95% CI 1.1–2.5, *I^2^ =* 59%) per 100 person-years (Supplementary material online, *Figure S14*). Leave-one-out analysis showed that study by Ma et al. contributed significantly to the heterogeneity (Supplementary material online, *Figure S15*).

#### Need for mitral valve intervention

The incidence of mitral valve intervention was documented in four studies (454; mean follow up 7.8 ± 3.7 years*). The pooled incidence rate was* 1.2 (95% CI 0.7–2.1, *I^2^ =* 62%) per 100 person-years (Supplementary material online, *Figure S16*). Leave-one-out analysis did not show any study particularly influenced the pooled estimate (Supplementary material online, *Figure S17*).

#### Congestive cardiac failure development

Development of congestive cardiac failure was documented in three studies in 978 patients. The pooled incidence rate was 1.0 (95% CI 0.3–2.9, *I^2^ =* 89%) per 100 person-years (Supplementary material online, *Figure S18*). Leave-one-out analysis did not show any study particularly influenced the pooled estimate (Supplementary material online, *Figure S19*).

#### Risk of bias assessment

Quality assessment showed that most prevalence studies had moderate quality (Supplementary material online, *Tables S8* and *S9*). Lack of information on response rate, target population and small sample size were the main issues increasing the risk of bias. Definition of MVP on two-dimensional echocardiography was commented on in 58% of the studies. Sensitivity analysis including studies with a low risk of bias resulted in a prevalence of 2.15% (95% CI 1.67–2.78%, *I*^2^ = 95%). Among the 5 studies reporting MR progression and adverse clinical events, the distribution of overall study quality was 50, 38, and 13% for high, moderate, and low-quality studies, respectively (Supplementary material online, *Figure S20*).

## Discussion

In this meta-analysis, we sought to quantitively establish the overall prevalence of both non-syndrome and syndrome associated MVP, as well as the prevalence across different age groups, diagnostic echocardiographic criteria, and study design. We also addressed the probability of MR progression in MVP and the incidence of adverse events.

The main findings were:

- The prevalence of MVP was estimated at 2.6%. In the general population, prevalence was 1.4%, but significantly higher in hospital cohorts (8.7%).
- Prevalence rates were markedly higher in patients with genetic syndromes: 57.2%, 18.4%, and 8.0% in Marfan's, Williams–Beuren, and Ehlers–Danlos syndromes, respectively.
- Prevalence was noted in infancy and increased with age, greatest in older-aged adults, at 2.9%.
- No significant gender-based differences were observed.
- Diagnostic criteria impacted prevalence, amplified by up to a factor of 4 with less specific criteria.
- The probability of MR progression by at least one grade was 5.5 per 100 person-years, overall and twice as fast from baseline moderate to severe MR.
- The incidence rate of all-cause mortality was 1.7 per 100 person years, while heart failure development and need for mitral valve intervention were 1.0 per 100 person-years.

The overall pooled prevalence of MVP was estimated at 2.6%, across diverse populations worldwide. This surpasses the general population estimate from our meta-analysis of 1.4%. The higher prevalence likely reflects the inclusion of subgroups with higher MVP rates, such as patients with scoliosis requiring surgery (12–24%)^26–28^ and individuals with polycystic kidney disease (1–26%).^13,29–31^ Methodological differences, particularly in patient selection partly account for the heterogeneity across studies.

Community-based studies employing stringent diagnostic criteria estimated a general population prevalence of 1.4% (0.81–2.23%), yet heterogeneity persisted. Subgroup analysis showed no gender-based differences. Genetic factors and variable disease penetrance may underlie the variability, as these remain poorly understood.^14^

The role of ethnicity is also unclear. The Strong Heart Study reported a prevalence of 1.7% in American Indians,^7^ aligning with our general population estimate, while the Framingham study found a higher prevalence of 2.4%,^1^ in predominantly white Americans. Similarly, a retrospective study in North East London observed higher MVP prevalence among white patients with primary MR,^32^ whereas the SHARE study in a multi-ethnic population, found a prevalence of 2.7% across all ethnicities.^33^

The exact mechanism for the development of MVP is unknown and may not be unitary.^14^ Our findings corroborate current evidence that MVP is significantly more prevalent in syndromes like Marfan's (57.2%), Williams (18.14%), and Ehlers–Danlos (8.0%), all of which have a discernible genetic component.^2,14^ While MVP has long been observed in Marfan's,^34^ Williams syndrome is more frequently associated with supravalvular aortic stenosis.^35^ Our findings suggest a higher prevalence of MVP than previously described and underscore the need for further research into its genetic and molecular determinants.

Our findings support a trend of increasing MVP prevalence with age, from 0.5% in neonates to 3.2% in middle-aged and older adults. This likely reflects the phenotypic distribution of MVP, with Barlow's disease detected in younger individuals and a rising incidence of fibroelastic deficiency in older adults.^2^ This contradicts previous work suggesting MVP's rarity in neonates and children.^11,12^ We estimated 1.8% of children express MVP, similar to Caglayan et al.,^36^ who reported a prevalence of 1.25% in Turkish school children (5–8 years), using stringent criteria. Ohara et al.,^11^ reported 2.5% using less specific criteria; and likely captured some non-diagnostic MVP cases. Whether such morphologies progress to diagnostic MVP as observed in adults remains unclear, highlighting the need for longitudinal studies.^6^ The adolescent group showed a surprisingly high prevalence of 2.7%, which may be transient and linked to the growth spurt,^37,38^ but it remains unclear whether this observation is due to enriched population sampling, looseness of diagnostic criteria, or a genuine feature of adaptive growth.

The influence of diagnostic methods on MVP prevalence has been noted.^2,5^ Older studies report higher prevalence rates, largely due to the use of low specificity M-mode echocardiography,^39,40^ while 2D echocardiography, the current standard, shows varying prevalence depending on the echocardiographic view. This review highlights that of stricter diagnostic criteria for MVP have a big role in defining disease prevalence, and that non-uniform criteria across studies have led to a high heterogeneity in the results. Diagnosing MVP by inspecting leaflet displacement in any view resulted in an estimated prevalence almost four times higher compared to more strict criteria defined by >2 mm leaflet displacement in the PLAX view (7.49 vs. 1.51%). The non-planarity of the mitral valve may explain this discrepancy, underscoring the importance of the PLAX view for diagnostic accuracy.^4^ However, whether more stringent criteria capture all patients with a prolapse phenotype remains unclear. Importantly, applying these criteria to young children is challenging due to their smaller heart size.

While most patients with MVP have no or mild MR, about 10% develop severe MR.^1^ Our meta-analysis found the probability of MR progression by at least one grade to be at 5.5 per 100 person-years, more than twice the rate observed in rheumatic heart disease, with an incident progression of 2.4 per 100 person-years.^41^ Moderate disease progressed significantly faster towards severe MR, at nearly 12 per 100 person-years compared to 1.5 per 100 person-years for mild disease. There is considerable variability between studies and non-uniform MR grading across studies encompassing qualitative, semi-quantitative, and quantitative methods may explain these differences.^6,18,42,43^ Nonetheless, moderate MR poses a higher risk of progression, emphasizing the need for closer monitoring. Factors other than severity, such as increasing mitral annulus diameters, chamber remodeling, and replacement fibrosis, may also be important, but this lies outside the scope of this investigation.^42,44–46^ Valve intervention is the only treatment for MVP and our findings indicate a low need for mitral valve intervention at 1.1 per 100 person-years. This shows that despite the progressive nature of MR in MVP, most patients do not meet current intervention criteria. While this suggests a generally favourable prognosis, only severe MR qualifies for intervention, according to current guidelines but moderate MR has been reported to incur excess mortality.^47^

The observed all-cause mortality rate of 1.7 per 100 person-years, is about four times higher than in the general population, estimated at 0.45 per 100 person-years in a 45–54 age group in a North American population,^48^ indicating a heightened mortality rate in individuals with MVP, not fully explained by heart failure development and the need for mitral valve intervention. Although the cause of the excess mortality could be due to the heterogeneity of the evidence, individual studies provide valuable insights. Aviernos et al., classified (using non-standard terminology) MVP patients as having primary (reduced ejection fraction, moderate/severe MR), and secondary (age >50, mild MR, left atrial enlargement, atrial fibrillation, and flail leaflet) risk factors, finding no excess mortality in low-risk patients (no primary risk factors) but elevated rates in those with multiple factors.^19^ Likewise, Ma et al., reported a lower mortality rate of 0.54 per 100 person-years in an asymptomatic population free of co-morbidities.^42^ While unlikely to fully explain the difference in mortality, these studies do emphasize the importance of conventional risk factors in prognostication.^19,47^ Importantly, in this study, we removed populations with known or suspected arrhythmic MVP at source (as typified by the mitral annular disjunction and systolic curling), considered a potentially distinct phenotype,^23^ so this signal comes from a more general population. These signs can be subtle and are a more recent phenotypic description so may still have been included, and we cannot exclude that some cases could relate to sudden cardiac death.^20^ Our ability to conduct an analysis on predictive factors was limited by the scarcity of data. Ultimately, these findings underscore the need for the design of risk stratification tools to guide clinical management and improve patient outcomes.

## Limitations

Phenotypic delineation of prevalence, namely fibroelastic insufficiency vs. Barlow's disease and classic vs. non-classic MVP could not be assessed, possibly explaining part of the high heterogeneity amongst studies. Some of the included studies failed to specify the identification technique utilized for MVP diagnosis which as demonstrated, generates significant variability in prevalence estimates. Meta-regression analysis for prevalence was constrained by data availability, limiting it to two covariates. MR progression, all-cause mortality and heart failure development couldn't undergo meta-regression due to the limited number of studies. This means conclusions on predictive factors could not be drawn. The sample size for adverse events was small and thus may be underpowered. Although the findings demonstrate MVP may develop at young ages, MR progression and rate of adverse events was only assessed in an adult population and therefore findings cannot be extended to an exclusively young adult and paediatric population. Moreover, comparison of adverse outcomes between the general population and syndromes was not conducted and should be assessed in a future study. Finally, outcome findings are only true for an intermediate follow up and highlight the need for longer term studies. We cannot determine in this investigation what the strength of the genetic elements of the disease are in those patients without a known clinical syndrome; the balance between the Barlow's phenotype and fibroelastic insufficiency (although this can be inferred from age) and the aetiology of the excess mortality we have observed.

## Conclusion

Designing services to address the care for patients with or at risk of MVP are complicated by a lack of clarity and incompleteness of the literature. In this report, we have demonstrated that MVP begins in infancy and increases through childhood with a higher prevalence in adults and especially older adults. Those with connective tissue genetic syndromes are at many times more additional risk which makes a case for very careful detection programmes in these populations. The choice of criteria for diagnosis alters the prevalence from 7.5 to 1.5% making this an important factor in the design of detection services, likewise hospital-based populations are clearly ‘enriched’ meaning the community prevalence is lower than that quoted in many studies. We have demonstrated both a substantial progression rate especially for those with moderate MR and a concerning signal of excess mortality for which the cause is unclear. It is imperative that valve services are designed to manage the whole life journey and the potential risk associated with MVP.

## Supplementary Material

qcaf016_Supplemental_File

## Data Availability

All data are incorporated into the article and its online supplementary material.
