# Supplementary material for: Prevalence, progression, and clinical outcomes of mitral valve prolapse: a systematic review and meta-analysis
Source: Eur Heart J Qual Care Clin Outcomes. 2025 Mar 28;11(5):631–41. doi: 10.1093/ehjqcco/qcaf016 (PMC12342860; doi:10.1093/ehjqcco/qcaf016)
Supplement: qcaf016_Supplemental_File [file qcaf016_supplemental_file.docx]

***Supplemental material***

**Methods**

**Search strategy**

**Prevalence**

(Mitral Valve Prolapse[MeSH Terms] OR "prolapse* mitral"[tiab:~3] OR "floppy mitra"[tiab:~3]

OR(mitral valve prolapse[Text Word] OR myxomatous valve disease[Text Word] OR diffuse

myxomatous degeneration[Text Word] OR Barlow* disease[Text Word] OR floppy valve[Text Word]))

AND (Incidence[MesH Terms] OR Prevalence[MesH Terms] OR Epidemiology[MesH Terms] OR

inciden*[Title/Abstract] OR prevalen*[Title/Abstract] OR epidemiol*[Title/Abstract])

**Progression**

(((Mitral Valve Prolapse[MeSH Terms] OR "prolapse* mitral"[tiab:~3] OR "floppy mitra"[tiab:~3]

OR(mitral valve prolapse[Text Word] OR myxomatous valve disease[Text Word] OR diffuse

myxomatous degeneration[Text Word] OR Barlow* disease[Text Word] OR floppy valve[Text Word]))

AND (Mitral Valve Insufficiency[MesH Terms] OR "regurgitation mitral"[tiab:~3] or "incompeten*

mitral"[tiab:~3] or "insufficien* mitral"[tiab:~3]) AND ((Risk[MeSH Terms] OR disease

progression[MeSH Terms] OR Morbidity[MeSH Terms] OR Mortality[MeSH Terms] OR

Prognosis[MeSH Terms]) OR risk*[Text Word] OR prognosis[Text Word] OR morbid*[Text Word] OR

mortal*[Text Word] OR outcome*[Text Word] OR "progression disease"[tiab:~2]))

**Adverse events**

((Mitral Valve Prolapse[MeSH Terms] OR "prolapse* mitral"[tiab:~3] OR "floppy mitra"[tiab:~3] OR(mitral valve prolapse[Text Word] OR myxomatous valve disease[Text Word] OR diffuse myxomatous degeneration[Text Word] OR Barlow* disease[Text Word] OR floppy valve[Text Word])) AND (("progression adj2 disease"[ti:~2]) OR (risk*[Title] OR prognosis[Title] OR morbid*[Title] OR

mortal*[Title] OR outcome*[Title]))))

**Data synthesis**

Total person follow-up years was calculated by multiplying the number of events, including the number of patients exhibiting MR progression or an adverse clinical event by the corresponding study’s follow up duration in years. The ratio between the number of events and the total person-years was used to determine event rates per 100 person years.

| Table 1: Baseline characteristics of included studies on non-syndrome associated mitral valve prolapse prevalence  **Author, year** | **Country** | **Type of study** | **Population** | **No.**  **patients** | **No.**  **Patients with MVP**  **n (%)** | **Mean age, years** | **Females (%)** | **Criteria for MVP diagnosis** |
| --- | --- | --- | --- | --- | --- | --- | --- | --- |
| Parvar, 2023^1^ | Iran | Retrospective | Neonates with suspected CHD | 6307 | 709 (11.2) | 8.5 ± 9.3 days | NR | NR |
| Dias 2022^2^ | Portugal | Retrospective | Athletes (<35yrs) undergoing CV examination for pre participation screening | 1981 | 5 (0.25) | NR | NR | NR |
| Savis 2022^3^ | UK | Retrospective | Polycystic kidney disease | 102 | 1 (0.98) | 10.3 ± 5.3 | 8.3 | One or both mitral valve leaflets bowed back into the left atrium and broke the plane of the MV annulus during systole from the parasternal long-axis view only. |
| Pfeferman 2022^4^ | Brazil | Retrospective | Polycystic kidney disease | 294 | 10 (3.4) | 41.0 ± 13.8 | 59.2 | Systolic displacement of the mitral leaflet into the LA at least 2mm from the mitral annular plane. |
| Fu 2021^5^ | Taiwan | Cross-sectional | School children | 769687 | 22988 (3.0) | 6-7 | NR | NR |
| Çağlayan 2021^6^ | Turkey | Cross-sectional | School children | 2550 | 32 (1.25) | 11.1 ± 2.9 | 52.5% | ≥2 mm displacement of the anterior and/or posterior mitral leaflet in at least the parasternal long axis view + diffuse mitral valve and chordal thickening. |
| Liu (CHIEF et) 2021^7^ | Taiwan | Cross-sectional | Military adults | 2442 | 82 (3.36) | 18-39 | 11.8% | Anterior/posterior displacement of the mitral valve leaflet to the mid portion of the annular hinge point > 2 mm in the parasternal long‐axis view. |
| Alsaady 2021^8^ | Iraq | Retrospective | Symptomatic patients visiting hospital cardiology outpatient department | 1456 | 64 (4.40) | 5-75 | 58.3 | Superior displacement of mitral valve leaflets 2mm or more towards the LA cavity during systole. |
| Modaff 2019^9^ | USA | Retrospective | Collegiate athletes | 2898 | 24 (0.8) | 18.8 | 51 | NR |
| Lang 2019^10^ | China | Retrospective | Idiopathic scoliosis requiring surgical treatment | 531 | 62 (11.68) | 17.8 ± 7.3 | 66.1 | NR |
| Rwebembera 2018 | Uganda | Retrospective | Adults with echo reports at a tertiary care centre | 15009 | 23 (0.15) | >13 | 62.9 | NR |
| Ong 2017^11^ | Canada | Cross-sectional | National hockey league athletes | 592 | 3 (0.2) | 18 | 0 | NR |
| Azami 2017^12^ | Iran | Cross-sectional | Patient referred to an outpatient cardiology clinic | 600 | 27 (4.5) | 44.1 ± 15.3 | 61.2 | ≥ 2mm cusp movement above the level of the mitral annulus |
| Bozcali, 2016^13^ | Turkey | Retrospective | Patients with scoliosis who underwent surgical correction. | 90 | 22 (24.4) | 14 ± 7 | 51 | NR |
| Kang 2015^14^ | China | Cross-sectional | Healthy school children | 2193 | 12 (0.54) | 5-13 | 47 | NR |
| Turker (MELEN) 2015^15^ | Turkey | Cross-sectional | Turkish adults | 2228 | 8 (0.36) | 49 ± 15 | 63.9 | Displacement measured in the parasternal long axis view above a line connecting the mid portions of the annular hinge points. |
| Nishio 2015^16^ | Japan | Cross-sectional | Children who underwent echo in local community events | 8819 | 5 (0.06) | 0-6 | NR | NR |
| Delling 2015  Framingham heart study: generation 3 cohort^17^ | USA | Prospective | Unselected community population | 3679 | 49 (1%) | 40 ± 9 | 53 | Leaflet displacement >2mm beyond the mitral annulus in a parasternal or apical long-axis view at end systole. |
| Güvenç 2012^18^ | Turkey | Cross-sectional | Residents living at a moderately high attitude | 494 | 30 (6.07) | 51±16.6 | 56 | ≥ 2 mm displacement of either or both MV leaflets superior to the mitral annular plane. |
| Rizzo 2012^19^ | Italy | Cross-sectional | Young male soccer players | 3100 | 10 (0.32) | 11 | 0 | NR |
| Tagarakis 2012^20^ | Greece | Prospective | Preventative cardiology outpatient clinic | 10818 | 238 (2.2) | 6-67 | NR | Systolic billowing of one or both mitral valve leaflets (>2mm) into the left atrium. |
| Ishikawa 2011^21^ | Japan | Cross-sectional | Newborns | 2067 | 4 (0.19) | 2.7±1.0 days | NR | NR |
| Liu 2011^22^ | China | Retrospective | congenital scoliosis admitted for surgical correction | 475 | 34 (7.2) | 12.8±5.8 | 39.7 | NR |
| Sattur 2010^23^ | USA | Retrospective | Teenagers undergoing screening echo | 2072 | 14 (0.7) | 13-19 | 33 | Single or bileaflet prolapse at least 2 mm beyond the long axis with or without leaflet thickening in the long-axis parasternal view and other views. |
| Hepner 2008^24^ | USA | Retrospective | Cardiac screening of teenage athletes | 1742 | 16 (0.9) | NR | 32.7 | NR |
| Strader 2008^25^ | USA | Retrospective | United States air force (USAF) pilot applicants | 20208 | 48 (0.24) | 23.6±2.5 | NR | NR |
| Hepner 2007^26^ | USA | Retrospective | Adults undergoing echo for a clinical reason | 24265 | (0.6) | 12-100 | 53 | NR |
| Bar-Dayan 2007^27^ | Israel | Retrospective | 17-year-old Israeli nationals | 94805 | 319 (0.34) | 17 | 38.5 | NR |
| Devereux 2001^28^ | USA | Cross-sectional | American Indians (Strong heart study) | 3340 | 57 (1.7) | 47-81 | 62.2 | Billowing of one or both mitral leaflets across the annular plane in 2D parasternal long axis recordings. |
| Balderas, 2001^29^ | Mexico | Retrospective | Adult and juvenile onset AS | 51 | 1 (2.0) | NR | NR | NR |
| Flack (CARDIA) 1999^30^ | USA | Retrospective | Black and white community young adults | 4136 | 26 (0.6) | 23-35 | 51.2 | 2mm or more of arching of either leaflet into the left atrium in the parasternal long axis view |
| Freed 1999 (Framingham heart study offspring examination 5)^31^ | USA | Retrospective | Unselected community population | 3491 | 84 (2.4) | 54.7±10.0 | 52.9 | >2mm superior displacement of the mitral leaflets during systole relative to the line connecting the annular hinge points in the parasternal and apical long axis views. |
| Hossack 1998^32^ | USA | Retrospective | Polycystic kidney disease | 163 | 42 (26) | 40±1 | 55 | 2D evidence + mitral click or M-Mode evidence, auscultatory evidence or doppler showing mitral incompetence. |
| Dhuper 1997^33^ | USA | Cross-sectional | Adolescents with severe idiopathic scoliosis requiring surgical treatment | 139 | 19 (13.6) | 13.8±0.45 | 79 | Excursion of one or both leaflets superior to the plane of the annulus in the parasternal long axis view on 2D Echo and confirmed by M-Mode. |
| Nascimento 1996^34^ | Portugal | Cross-sectional | Newborns | 1734 | 0 | 4hrs -7 days | NR | Posterosuperior motion of one or both mitral leaflets beyond the annular plane in the parasternal long axis view/ posterior leaflet in any view |
| Kahaly 1995^35^ | Germany | Cross-sectional | Lymphocytic Autoimmune Hashimoto’s thyroiditis | 50 | 18 (36) | 19-67 | 80 | Systolic displacement into the left atrium of one or both mitral leaflets in the parasternal long axis view |
| Ivy 1995^36^ | USA | Cross-sectional | Children with polycystic kidney  disease | 154 | 10 (12) | 1.5-17 | NR | >2mm posterior systolic displacement of the mitral valve in two views or more by 2D TTE. |
| Gupta 1992^37^ | India | Cross-sectional | School children | 213 | 28 (13.1) | 3-12 | NR | Systolic prolapse of anterior, posterior or both mitral valve leaflets into the left atrium on parasternal long axis view. |
| Sharif 1991^38^ | UK | Cross-sectional | Keratoconus patients requiring corneal transplant | 50 | 29 (58) | 32 | 36 | Posteriorly displaced coaptation of the mitral leaflets OR a superior movement of either leaflet above the mitral ring. |
| Ohara 1991^39^ | Japan | Cross-sectional | Children | 4238 | 109 (2.6) | 1-15 | 50.4 | NR |
| Street 1991^40^ | USA | Cross-sectional | Keratoconus | 80 | 14 (17) | 19-72 | 38 | Posterior systolic bulging of the mitral leaflets above a line between the base of the aortic valve and the atrioventricular junction OR superior systolic displacement of either or both leaflets above the level of the mitral annulus on the 4-chamber view. |
| Ozeren 1998^41^ | Turkey | Retrospective | Bipolar affective disorder | 22 | 2 (9.09) | 42.3 | NR | Prolapse of the anterior or posterior mitral valve into the left atrium seen in 2D echo AND a mid-late systolic motion of mitral valve >2mm in M-Mode. |
| Holgado 1987^42^ | USA | Cross -sectional | Hypertension | 133 | 1(0.75) | 54±14 | 51.1 | Systolic arching of one or both MV leaflets posteriorly and superiorly into the left atrium in the parasternal long axis view/apical 4 chamber view or excessive posterior coaptation of MV leaflets. |
| Liberthon 1986^43^ | USA | Cross-sectional | Panic disorder | 131 | 34 (26) | 38 | NR | Systolic movement of any part of the anterior or posterior mitral valve leaflet in a plane superior to the mitral annulus in two echo views or bileaflet prolapse in a single view. |
| Kumaki 1985 | Japan | Cross-sectional | University students | 4517 | 42 (0.93) | NR | 21.0 | NR |
| Warth 1985 | USA | Cross-sectional | Children attending well childcare outpatient appointments. | 193 | 13% | 4.5 | 49.2 | superior systolic motion of at least one mitral valve leaflet above the mitral annular plane to be present in either the parasternal long axis view, 4-chamber or both. |
| Beardsley 1982 | USA | Cross-sectional | Keratoconus | 32 | 12 (37.5) | 27 | 44 | Posteriorly displaced coaptation of the mitral leaflets or a superior movement of either leaflet above the mitral ring during systole. |

MVP: Mitral valve prolapse; CV: Cardiovascular; LA: Left atrium; AS: Ankylosing spondylitis; NR: Not reported

| Table 2: Baseline characteristics of included studies on the prevalence of MVP in Marfan syndrome.  **Author/year** | **Country** | **Type of study** | **Population** | **No.**  **patients** | **No.**  **Patients with MVP**  **n(%)** | **Mean age, years** | **Females MVP (%)** | **Criteria for MVP diagnosis** |
| --- | --- | --- | --- | --- | --- | --- | --- | --- |
| Backer 2006^44^ | Belgium | Cross-sectional | Marfan syndrome who fulfilled Gent criteria | 53 | 35 (66) | 33.0±10.9 | 53 | Leaflet displacement exceeding 2mm from the parasternal long axis and 4 chamber view. |
| Espinola-Zavaleta 2010^45^ | Mexico | Prospective | Unselected Mestizo – Mexican MFS patients | 114 | 70 (61) | Children: 7.8±4.2  Adults: 32.5 ±9 | 54 | Displacement of either MV leaflet >2mm during systole relative to a line drawn across the annulus in the parasternal long axis and confirmed in the apical views. |
| Geva 1987 ^46^ | Israel | Retrospective | Children with Marfan syndrome | 25 | 25 (100) | 8.1 ± 4.8 | 16 | NR |
| Gu 2015 ^47^ | China | Retrospective | Marfan syndrome patients who underwent cardiovascular surgery | 73 | 15 | 32±12 | 29 | > 2mm billowing of one or more MV leaflet/s above the mitral annulus in the PLAX or apical long axis view. |
| Hirata 1992^48^ | USA | Cross-sectional | Maran’s syndrome diagnosed by strict criteria | 23 | 22 | 28.2 ± 8.6 | 33 | Either or both mitral leaflets bulged into the LA beyond the mitral annulus in the PLAX. |
| Karnebeek 2001^49^ | Netherlands | Retrospective | Young people with Marfan syndrome | 52 | 46 (88) | 7.9 (1.0 - 16) | 48 | Moderate-severe bowing of the anterior and/or posterior MV leaflet(s) into the LA with coaptation of the leaflets at the level, or on the atrial side of the mitral annulus. |
| Kunkala 2013^50^ | USA | Retrospective | Marfan’s syndrome who underwent aortic root replacement | 239 | 166 (69) | 32±13 | 71 | Leaflet thickness >0.5mm and leaflet motion >2 above the plane of the mitral annulus in the long-axis view. |
| Lopez 2015^51^ | Brasil | Prospective | Children with Marfan’s syndrome | 21 | 11(52) | 10 (median) | 38 | Superior displacement of one or both mitral leaflets by >2mm above the mitral plane during ventricular systole in the parasternal long axis view. |
| Mielczarek 2018^52^ | Poland | Cross sectional | MFS patients diagnosed according to the modified Ghent criteria | 101 | 55(54.5) | 23.8±15.3 | 46 | NR |
| Mueller 2013^53^ | Germany | Cross -sectional | Paediatric population with MFS | 82 | 26(31.7) | 9.66 ± 5.87 | 54 | Leaflet thickness exceeding 5mm and systolic prolapse of the leaflets into the left atrium >2mm. |
| Muhlstadt 2019 ^54^ | Germany (multicentre) | Retrospective | Fulfilling Ghent criteria for MFS | 83 | 48 (57.8) | 34 ±18 | 56 | >2mm superior displacement of the mitral leaflets during systole relative to the line connecting the annular hinge points in the parasternal and long axis views. |
| Ozdemir 2011^55^ | Africa | Cross -sectional | Young patients with MFS | 11 | 11 (100) | 4-15 yrs  Median 11 | 36.4 | Leaflet thickness >5mm and systolic prolapse of the leaflet(s) into the atrium of more > 2mm in the parasternal long axis and 4-chamber views. |
| Rybczynski 2010^56^ | Germany | Cross-sectional | Unselected population of MFS | 204 | 82 (40) | 31.2±16.4 | 47 | leaflet displacement >2mm from the parasternal long axis, 4 chamber view and M-mode. |
| Taub 2009^57^ | USA | Retrospective | MFS patients | 90 | 25 (28) | 33 ± 16 | 56 | displacement of either anterior or posterior mitral leaflets >2 mm in the parasternal long-axis view and confirmed in the apical long axis view. |
| Yetman 2003^58^ | Canada | Cross sectional elements | MFS patients diagnosed with revised criteria | 70 | 34 (49) | NA | NA | NA |

MVP: Mitral valve prolapse; CV: Cardiovascular; LA: Left atrium; NR: Not reported.

| **Author/year** | **Type of study** | **Country** | **Population** | **N patients** | **No.**  **Patients with MVP**  **n (%)** | **Mean age, years ± SD** | **Females (%)** | **Criteria for MVP diagnosis** |
| --- | --- | --- | --- | --- | --- | --- | --- | --- |
| Asher 2017^59^ | Cross-sectional | USA | HEDs, HSDs, CEDS > 15yrs | 209 | 13 (6.2%) | NR | 87.6 | NR |
| Atzinger 2011^60^ | Retrospective | USA | HEDs and CEDs | 252 | 15(6) | NR | NR | NR |
| Dolan 1997^61^ | Cross-sectional | UK | EDS | 33 | 2 (6.1%) | Median 35 (18-50) | 82 | i) Movement of the point of apposition of the mitral valve leaflets behind the plane of the annulus into the left atrium during systole, assessed in the apical 4 chamber view ii) movement of a part of either leaflet above the plane of the annulus into the LA in systole, assessed in the parasternal long axis view. |
| Foltz 2019^62^ | Retrospective | USA | HEDs or HSD | 208 | 2 (0.96%) | 12.0 ± 9.0 | 80 | NR |
| Forrest 1980^63^ | Prospective | USA | Patients meeting criteria for hypermobility | 23 | 2 | 27.8 | NR | NR |
| Piteri Toro 2023^64^ | Retrospective | USA | HEDs | 62 | 5(8.1%) | 32.7 ± 12 | 97 | NR |
| Rashed 2022^65^ | Retrospective | USA | HEDs or HSD | 258 | 19 (7.5%) | Median 31 (4-73) | 90 | NR |
| Shiari 2012 | Cross -sectional | Iran | HEDs | 63 | 34 (54%) | 7.1 ± 6.7 | NR | Mitral leaflet displacement >2mm |

Table 3: Baseline characteristics of included studies on the prevalence of MVP in Ehlers-Danlos syndrome

MVP: Mitral valve prolapse; HEDs: Hypermobile Ehlers-Danlos syndrome; CEDs: Classic Ehlers-Danlos syndrome; LA: Left atrium; NR: Not reported

Table 4: Baseline characteristics of included studies on the prevalence of MVP in Williams–Beuren Syndrome

| **Author/year** | **Type of study** | **Country** | **N patients** | **Patients MVP, n (%)** | **Mean age, years** | **Follow-up, years** | **Female (%)** | **Criteria for MVP diagnosis** |
| --- | --- | --- | --- | --- | --- | --- | --- | --- |
| Bruno 2023^66^ | Prospective | Argentina | 53 | 12 (27%) | 7.3±4.5 | NR | NR | Any or both mitral valve leaflets with systolic displacement above the mitral valvar annulus into the left atrium greater than 2mm. |
| Cha 2019^67^ | Retrospective | South Korea | 80 | 18 (22.5%) | At diagnosis: 1.0 (0-17.3) | 11.0 (5.1 ± 28.3) | 43 | Systolic displacement of total or a portion of the mitral valve beyond the plane of the mitral annulus in the parasternal long-axis view. |
| Collins 2010^68^ | Retrospective | USA | 270 | 40 (15%) | At diagnosis: 3.3± 5.9 | 8.9 (0-56.9) | 50 | NR |
| Ergul 2012^69^ | Retrospective | Istanbul | 45 | 10 (22.2%) | At diagnosis 4.6±3.1 | 6.9 ±4.4 | 40 | Parasternal long axis view if either leaflet of the valve had >2mm systolic displacement above the annulus in the left atrium. |
| Lee 2022^70^ | Retrospective | Taiwan | 30 | 9 (30) | Median 11.3 (0-48) | 5.6 (0.1-12.8) | 37 | NR |
| Pasqua 2009^71^ | Retrospective | Italy | 150 | 7 (6.2) | 13.9 (7 month-45yrs) | NR | 55 | Parasternal long axis view if either leaflet of the valve had >2mm systolic displacement above the annulus in the left atrium. |
| Scheiber 2006^72^ | Retrospective | Hungary | 29 | 9 (31.0) | 12.8 | NR | 59 | Prolapse >2mm in two different planes and/or mitral valve thickening and typical fibrillation detected during opening. |
| Smith 1988^73^ | Cross-sectional | UK | 61 | 9(15) | 9.8 (8-29) | NR | 53 | NR |

MVP: Mitral valve prolapse; NR: Not reported.

Table 5: Baseline characteristics of included studies reporting MR progression and/or adverse events

| **Author, year** | **Country** | **Type of study** | **Population** | **N**  **Patients** | **Mean age, years** | **Females (%)** | **Posterior prolapse n (%)** | **Bileaflet prolapse n (%)** | **Mild MR baseline** | **Moderate MR baseline** | **LVEF (%)** | **Follow up, years** |
| --- | --- | --- | --- | --- | --- | --- | --- | --- | --- | --- | --- | --- |
| Ma 2019^74^ | USA | Retrospective | Asymptomatic MVP and mild or moderate MR | 82 | 65±12 | 49 | NR | NR | 36 (44) | 46  (54%) | 61.9 ± 7.7 | 4.5±2.7 |
| Delling 2016^75^ | USA | Retrospective | Unselected community population | 63 | 57 ± 11 | 52 | 28 (44%) | NR | NR | NR | NR | Progression: 11  Events: 19 ± 4 |
| Aviernos 2008 ^76^ | USA | Prospective | Unselected community population | 285 | 56 ± 22 | 57 | 56 (24) | 90(38) | 35 (12) | 43(15) | 60 ± 9 | 4.6±2.9 |
| Wand 2011^76^ | Israel | Prospective | Military aircrew aviators | 24 | 17.9± 4.1 | 0 | 10(41.7) | 9(37.5) | 6 (27.3) | NR | NR | Progression: 10.9  Events: 23.5 |
| Aviernos 2002^81^ | USA | Prospective | Echo for a clinical reason | 833 | 50±21 | 64 | 34 | 39 | 46 | NR | 62 ± 7 | 5.4 median |

**
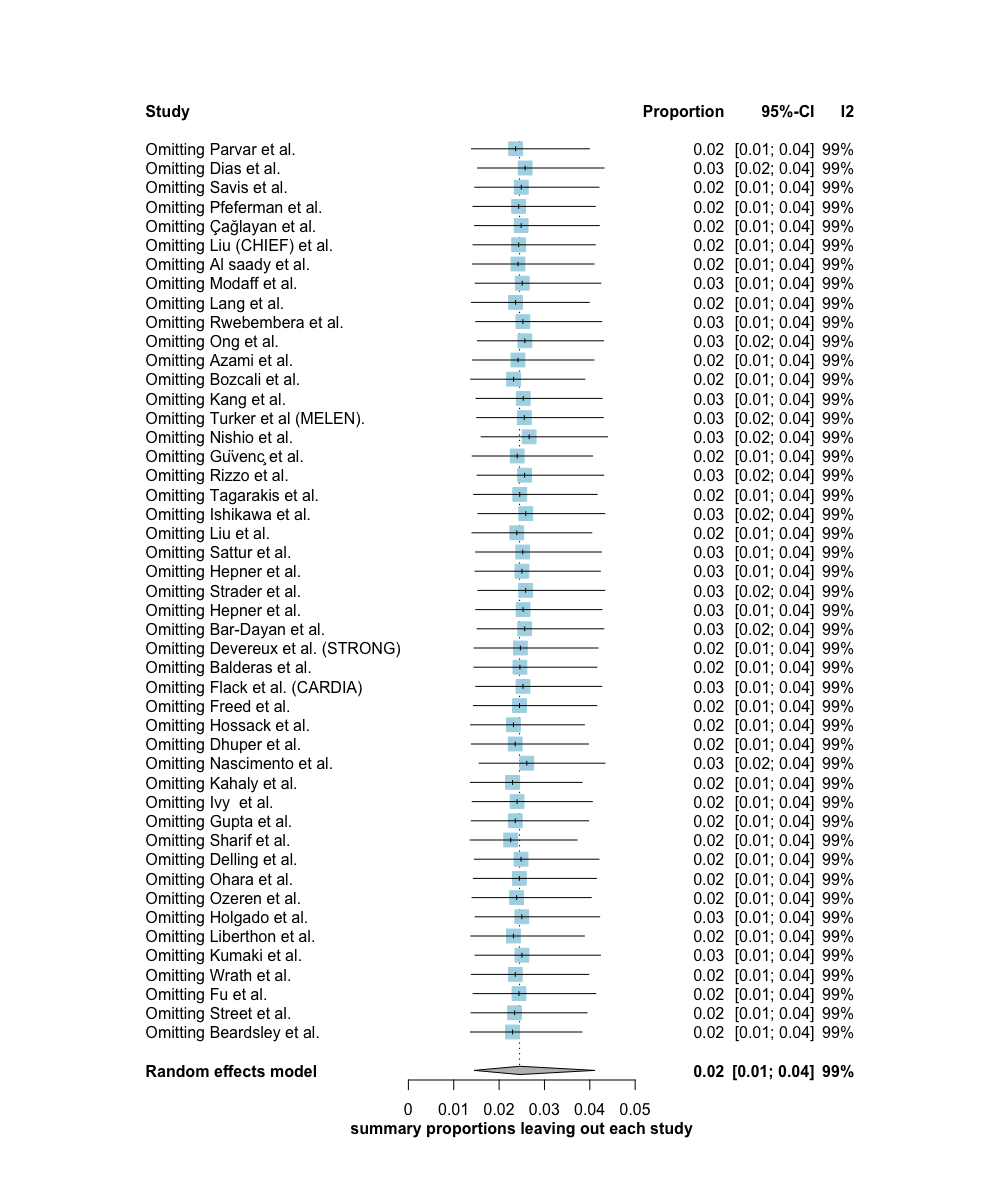
**

Figure 1: Leave-one-out sensitivity analysis for non-syndrome associated MVP prevalence


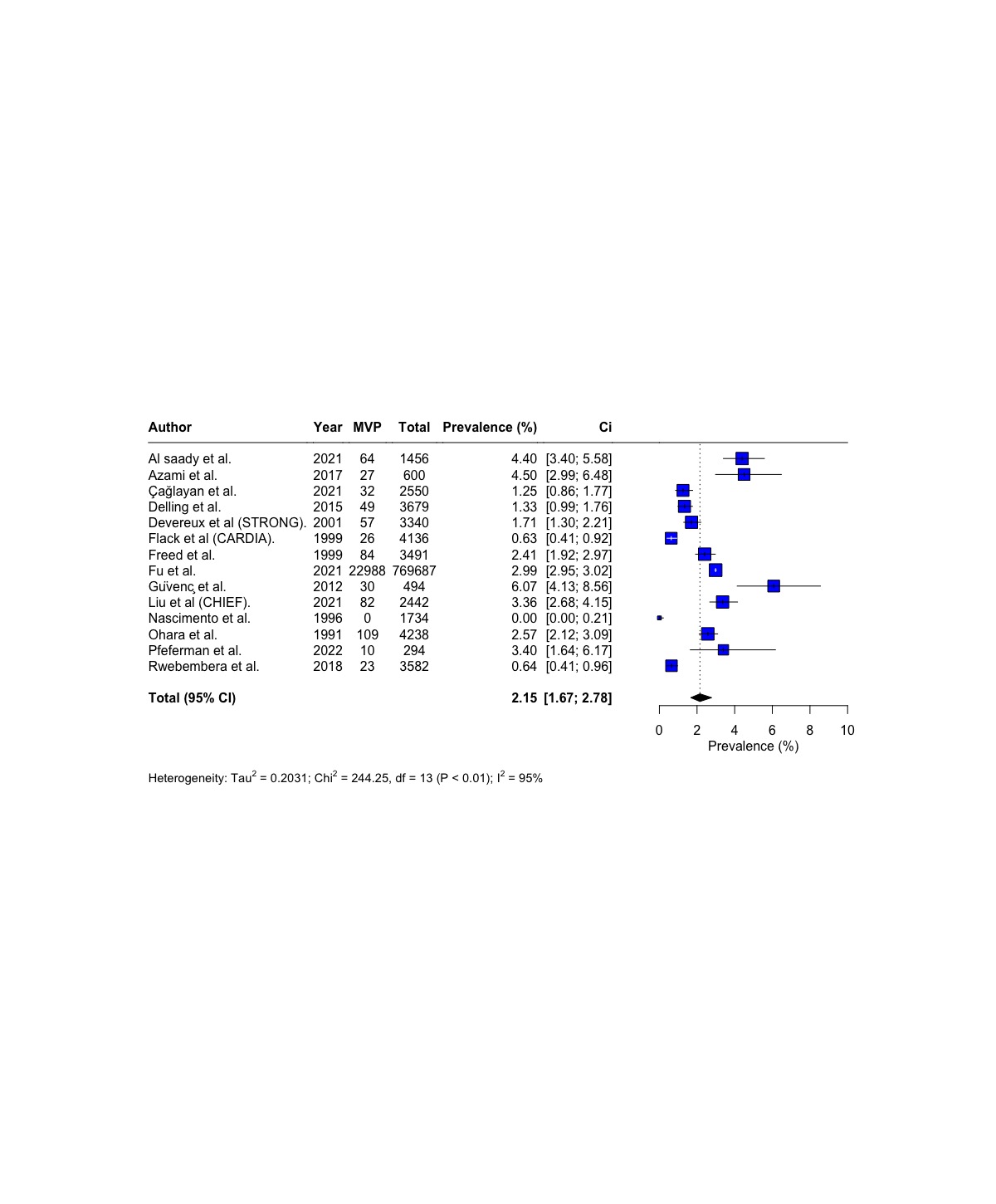


Figure 2: Forest plot showing MVP prevalence limited to studies with a low methodological risk of bias.

**
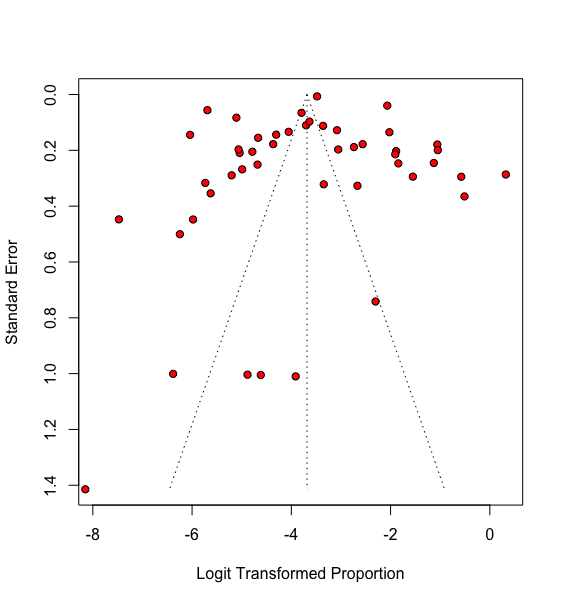
**

Figure 3: Funnel plot for non-syndrome associated MVP prevalence.

The funnel plot for risk of publication bias assessment appears symmetrical and alongside eggers regression test (p= 0.71), provide no evidence to suggest the presence of publication bias.

**Handling heterogeneity**

Sensitivity analysis, applying various transformations including generalised linear mixed model, logs and Freeman-Tukey double arcsine were conducted for the estimate of MVP prevalence in the general population and syndromes. This assessed the consistency of results across diverse analytical approaches.

Table 6: Sensitivity analysis results for non-syndromic MVP prevalence using diverse analytical techniques

| Transformation method | Pooled prevalence (%) | 95% Confidence Interval | I^2^ (%) |
| --- | --- | --- | --- |
| PLOGIT | 2.59 | 1.45-4.11 | 99 |
| Freeman-Tukey Double Arcsine | 3.55 | 2.70-4.51 | 100 |
| Log | 2.46 | 1.77-3.40 | 99 |
| GLMM | 2.27 | 1.32-3.86 | 99 |

The pooled prevalence was estimated at 3.55% (95% CI 2.70-4.51%, I2 = 100%) when Freeman Tukey Double arsine transformation was applied, 2.46% (95% CI 1.77-3.40%, I2 = 99%) when applying a Log transformation and 2.27% (95% CI 1.32 – 3.86%, I2 = 99%) when the Generalized Linear Mixed Model (GLMM) formed the meta-analytical method.

*Figure 4: Forest plot displaying pooled prevalence among systematic screening community and population-based studies compared to hospital cohorts.*

*Figure 5: Forest plot displaying pooled prevalence among community-based studies stratified by sex.*


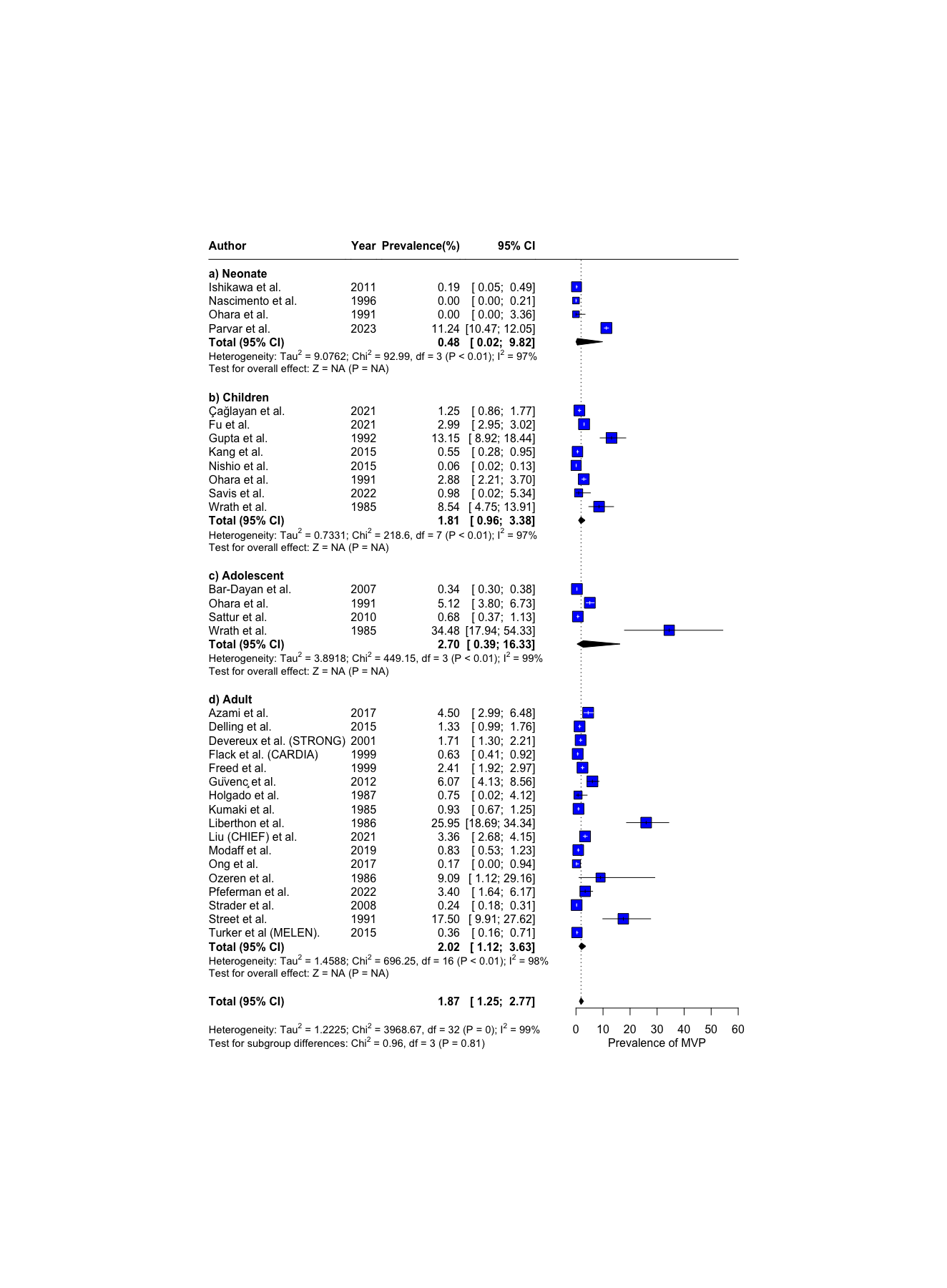


Figure 6: Age group sub-group analyses forest plot.


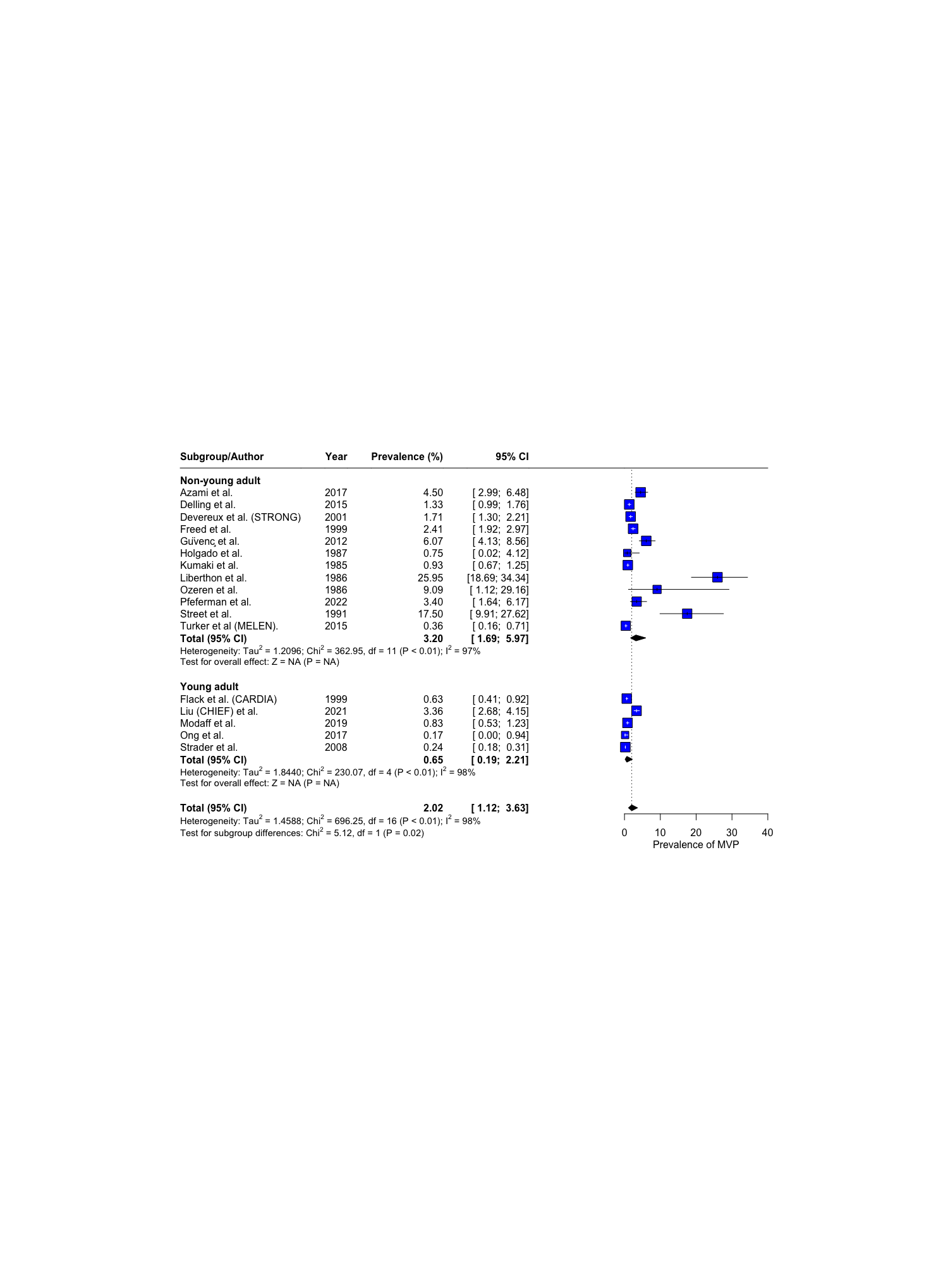


Figure 7: Adult vs non adult sub analyses forest plot.


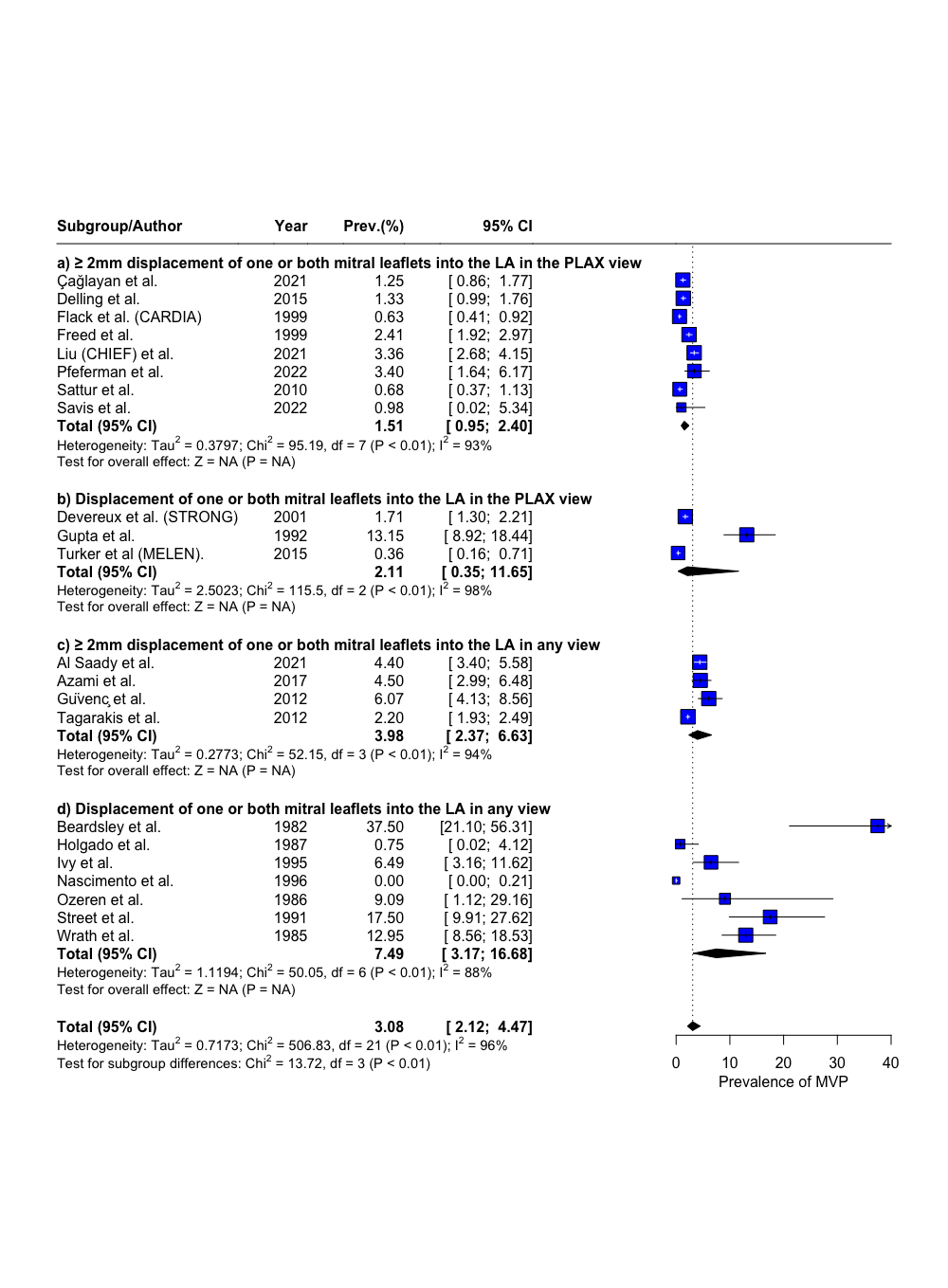


Figure 8: Forest plot showing prevalence based on echocardiography diagnostic criteria.


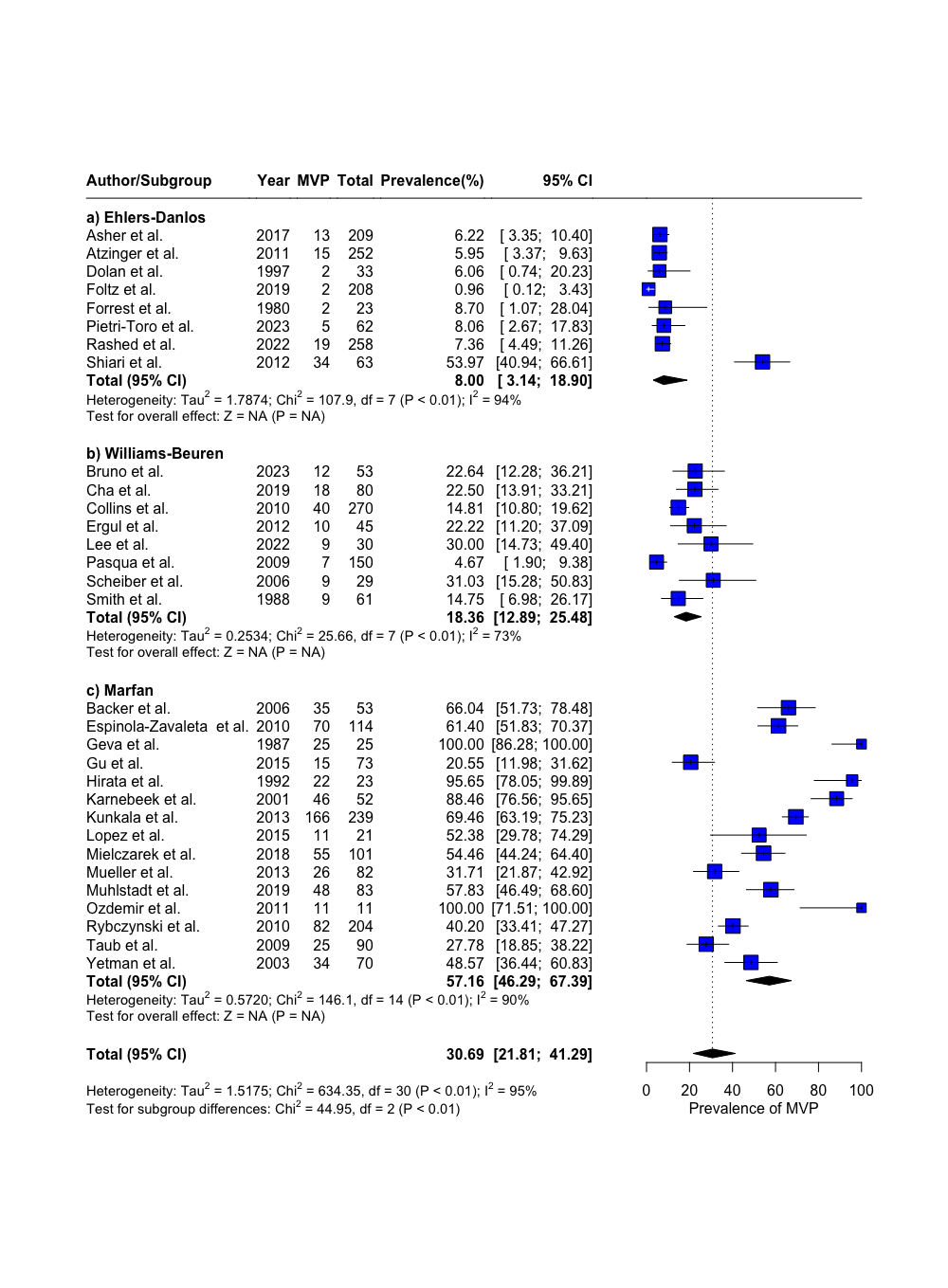


Figure 9: Forest plot for pooled MVP prevalence in syndromes.


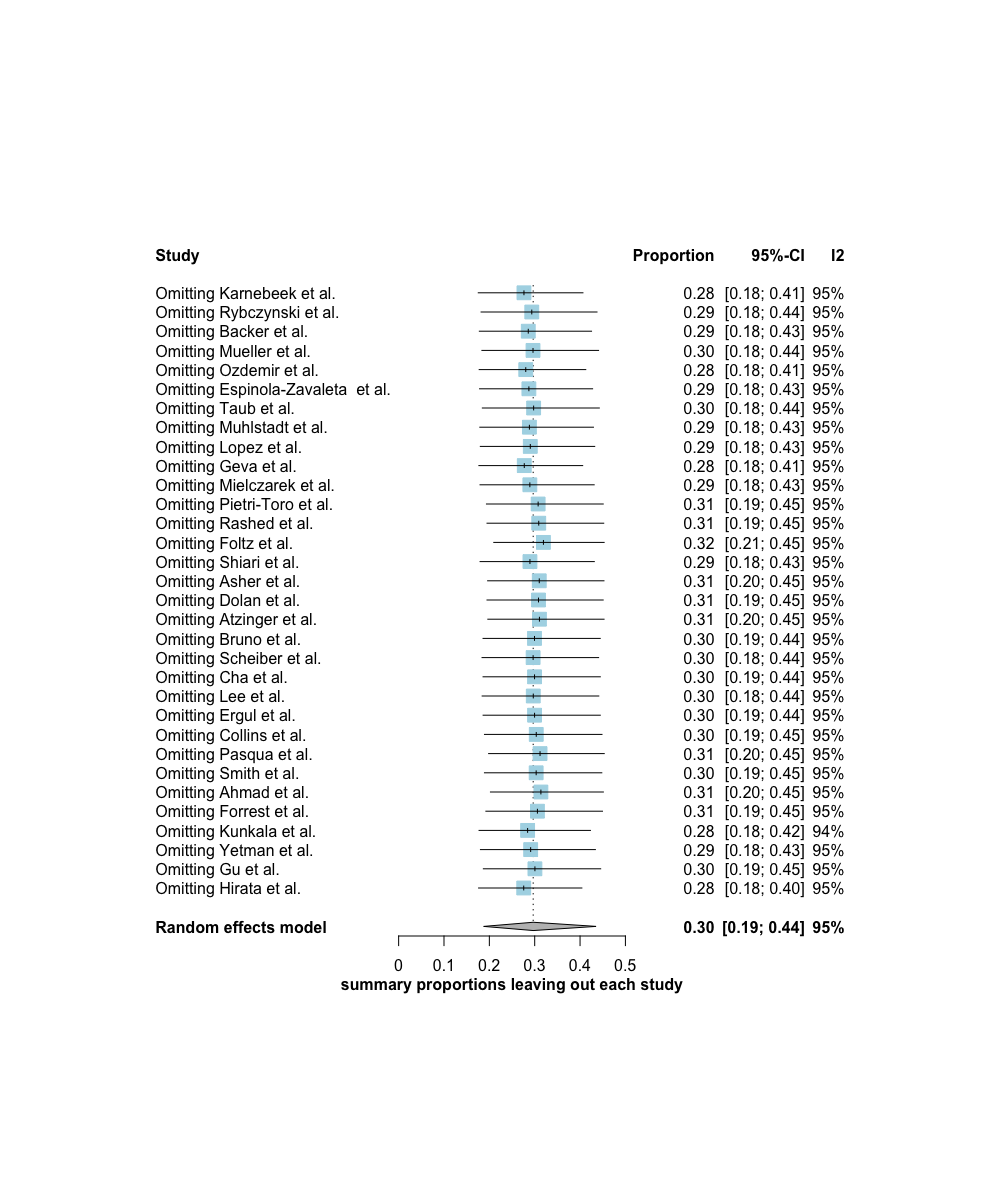
 Figure 10: Leave-one-out sensitivity analysis for syndrome associated MVP prevalence.


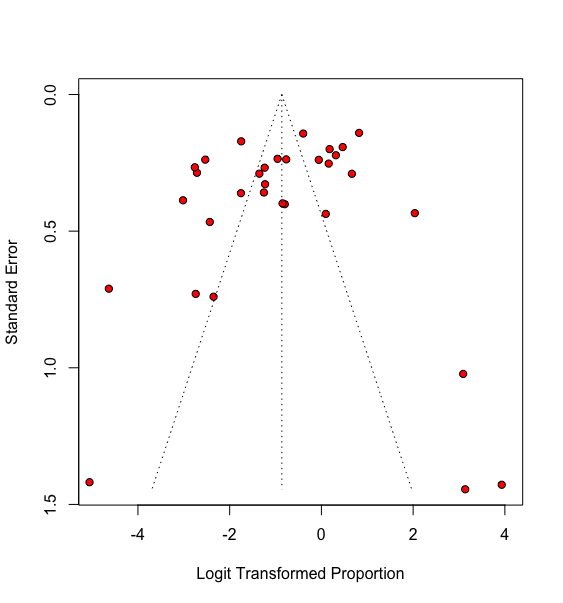


Figure 11: Funnel plot for syndromic MVP prevalence.

Table 7: Sensitivity analysis results for syndromic MVP prevalence using diverse analytical techniques.

Marfan syndrome

| Transformation method | Pooled prevalence (%) | 95% Confidence Interval | I^2^ (%) |
| --- | --- | --- | --- |
| PLOGIT | 57.16 | 4.63-6.74 | 90.4 |
| Freeman-Tukey Double Arcsine | 62.31 | 50.23- 73.69 | 94.1 |
| Log | 57.73 | 48.53-68.67 | 95.9 |
| GLMM | 65.20 | 47.17-79.73 | 89.6 |

Ehlers-Danlos

| Transformation method | Pooled prevalence (%) | 95% Confidence Interval | I^2^ (%) |
| --- | --- | --- | --- |
| PLOGIT | 8.00 | 3.14-18.90 | 93.5 |
| Freeman-Tukey Double Arcsine | 9.45 | 3.43-1.77 | 93.1 |
| Log | 7.45 | 2.82-19.65 | 95.7 |
| GLMM | 7.48 | 3.20-16.51 | 93.5 |

Williams-Beuren

| Transformation method | Pooled prevalence (%) | 95% Confidence Interval | I^2^ (%) |
| --- | --- | --- | --- |
| PLOGIT | 18.36 | 12.9-35.48 | 72.7 |
| Freeman-Tukey Double Arcsine | 18.27 | 11.76-25. 77 | 79.7 |
| Log | 18.56 | 13.35-25.79 | 72.3 |
| GLMM | 17.81 | 12.18-25.29 | 72.7 |


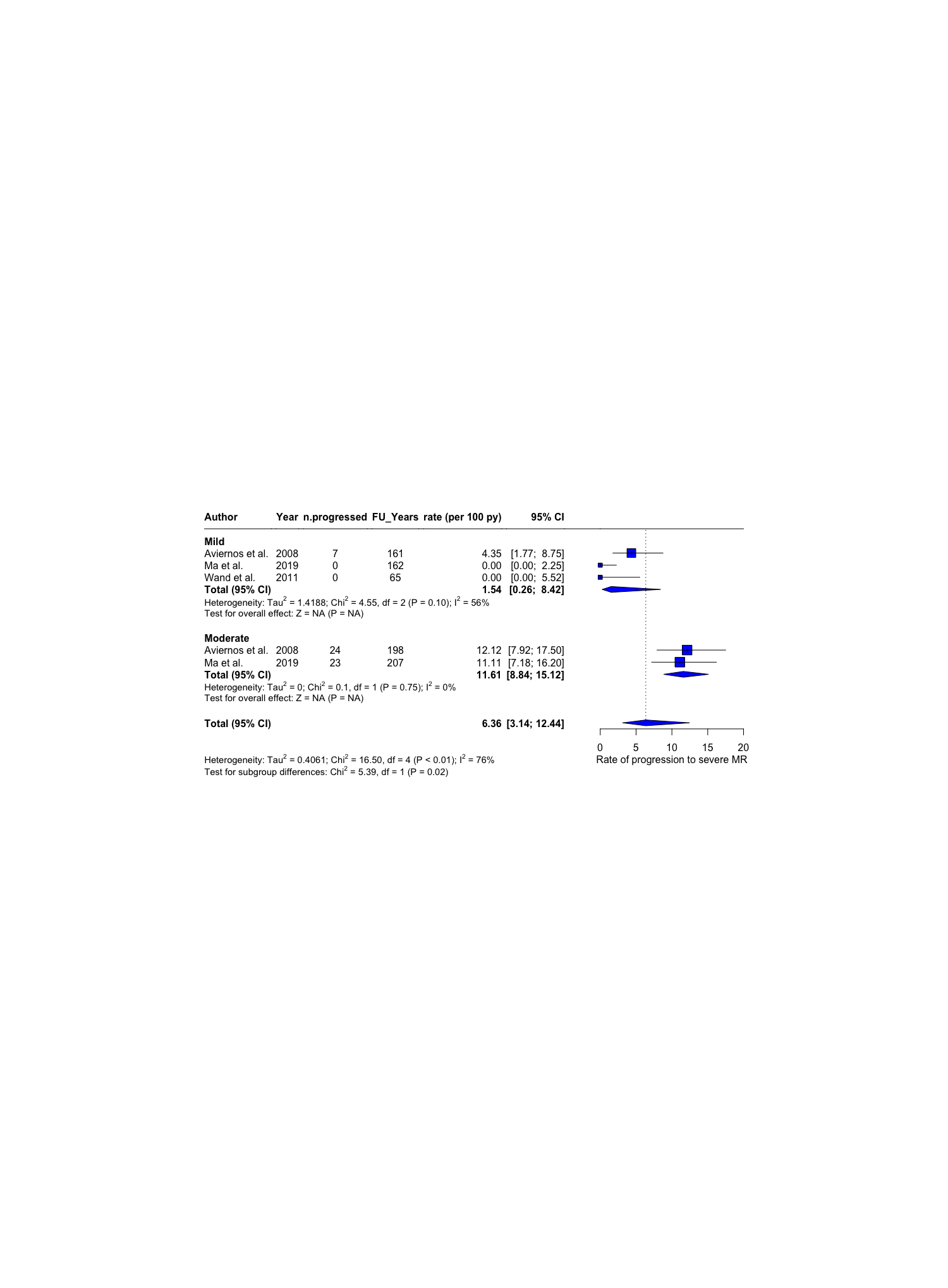


Figure 12: Forest plot for progression towards severe MR stratified by baseline MR severity.

Figure 13: Forest plot for MR progression based on study design (retrospective vs prospective).


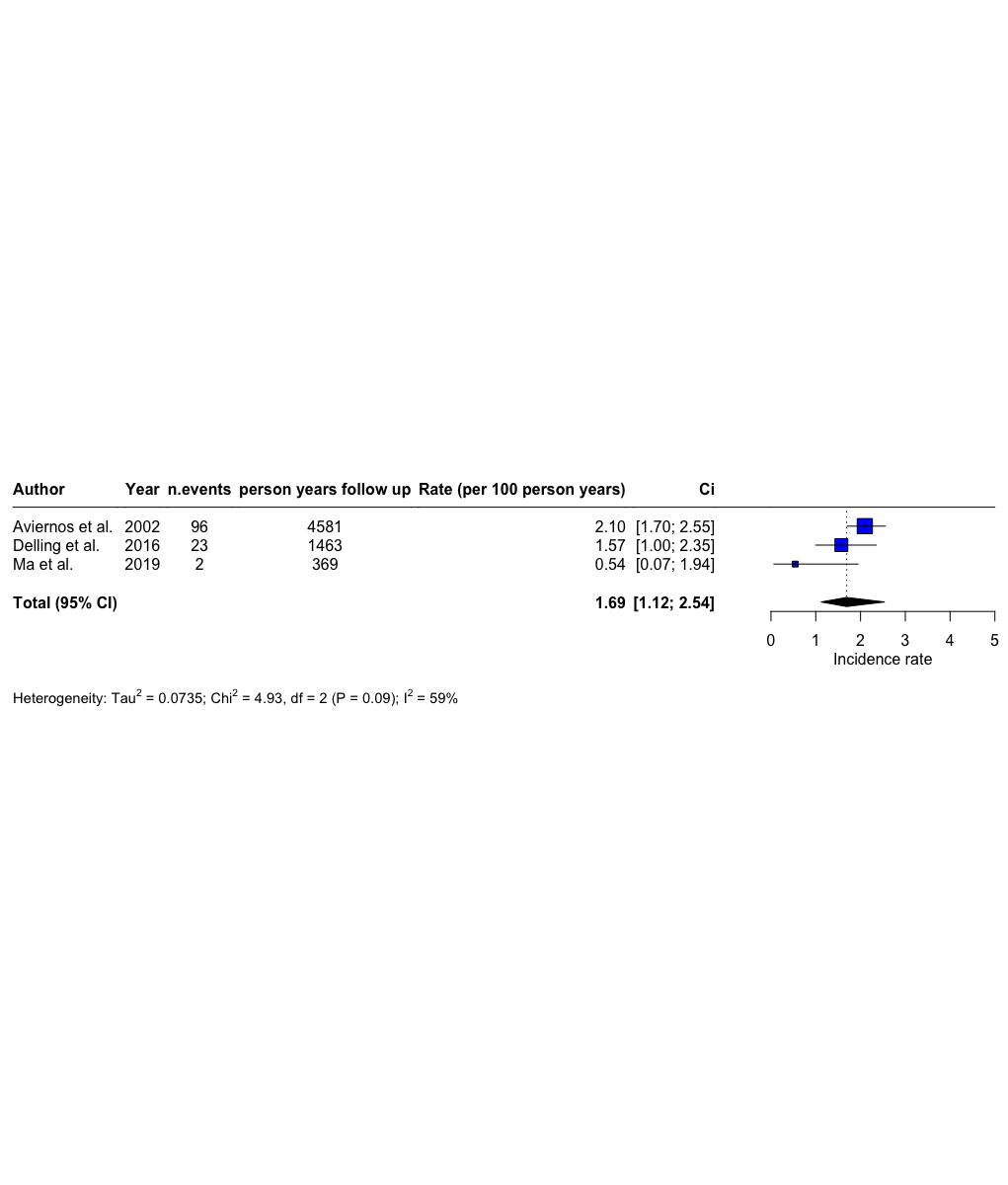


Figure 14: Forest plot for incidence rate of all-cause mortality.

Figure 15: Leave-one-out sensitivity analysis for all-cause mortality.

Figure16: Forest plot illustrating the incidence rate of mitral intervention.

Figure17: Leave-one-out sensitivity analysis for rate of mitral intervention

Figure 18: Forest plot for incidence rate of heart failure development.

*Figure 19: Leave-one-out sensitivity analysis for heart failure development*.

Table 8: Risk of bias assessment summary table for non-syndromic prevalence studies

| Study | Q1 | Q2 | Q3 | Q4 | Q5 | Q6 | Q7 | Q8 | Q9 | Overall |
| --- | --- | --- | --- | --- | --- | --- | --- | --- | --- | --- |
| Parvar, 2023 | N | Y | Y | Y | Y | U | Y | Y | NA | Moderate |
| Dias 2022 | N | Y | Y | Y | Y | U | N | Y | NA | Moderate |
| Savis 2022 | N | Y | N | Y | Y | Y | Y | Y | NA | Moderate |
| Pfeferman 2022 | N | Y | Y | Y | Y | Y | Y | Y | NA | High |
| Fu 2021 | Y | Y | Y | Y | Y | U | N | Y | Y | High |
| Çağlayan 2021 | Y | Y | Y | Y | Y | Y | Y | Y | NA | High |
| Liu (CHIEF) 2021 | N | Y | Y | Y | Y | Y | Y | Y | NA | High |
| Alsaady 2021 | N | Y | Y | Y | Y | Y | Y | Y | NA | High |
| Modaff 2019 | N | Y | Y | Y | Y | U | Y | Y | NA | Moderate |
| Lang 2019 | N | Y | Y | Y | Y | U | Y | Y | NA | Moderate |
| Rwebembera 2018 | N | Y | Y | Y | Y | Y | Y | Y | NA | High |
| Ong 2017 | N | Y | N | Y | Y | U | U | Y | NA | Moderate |
| Azami 2017 | N | Y | Y | Y | Y | Y | Y | Y | NA | High |
| Bozcali, 2016 | N | Y | N | Y | Y | U | Y | Y | NA | Moderate |
| Kang 2015 | N | Y | Y | N | Y | U | Y | Y | NA | Moderate |
| Turker (MELEN) 2015 | Y | Y | N | Y | Y | Y | U | Y | U | Moderate |
| Nishio 2015 | N | Y | Y | Y | Y | U | Y | Y | NA | Moderate |
| Delling 2015 | Y | Y | Y | Y | Y | Y | Y | Y | U | High |
| Güvenç 2012 | N | Y | Y | Y | Y | Y | Y | Y | NA | High |
| Rizzo 2012 | N | Y | Y | N | Y | U | Y | Y | NA | Moderate |
| Tagarakis 2012 | N | Y | Y | Y | Y | Y | U | Y | NA | Moderate |
| Ishikawa 2011 | N | Y | Y | N | Y | U | Y | Y | NA | Moderate |
| Liu 2011 | N | N | Y | Y | U | N | U | Y | NA | Low |
| Sattur 2010 | Y | N | Y | Y | Y | Y | U | Y | NA | Moderate |
| Hepner 2008 | N | N | Y | Y | Y | U | Y | Y | NA | Moderate |
| Strader 2008 | N | Y | Y | N | Y | U | Y | Y | NA | Moderate |
| Hepner 2007 | N | Y | Y | Y | Y | N | Y | Y | NA | Moderate |
| Bar-Dayan 2007 | N | Y | Y | Y | Y | U | U | Y | NA | Moderate |
| Devereux 2001 | N | Y | Y | Y | Y | Y | Y | Y | NA | High |
| Balderas, 2001 | N | Y | N | Y | Y | U | Y | Y | NA | Moderate |
| Flack (CARDIA) 1999 | Y | Y | Y | Y | Y | Y | Y | Y | N | High |
| Freed 1999 | Y | Y | Y | Y | Y | Y | Y | Y | Y | High |
| Hossack 1998 | N | Y | N | Y | Y | Y | U | Y | NA | Moderate |
| Dhuper 1997 | N | Y | N | Y | Y | Y | U | Y | NA | Moderate |
| Nascimento 1996 | Y | Y | Y | Y | Y | Y | Y | Y | NA | High |
| Kahaly 1995 | N | Y | N | Y | Y | Y | N | Y | NA | Moderate |
| Ivy 1995 | N | Y | N | Y | Y | Y | Y | Y | NA | Moderate |
| Gupta 1992 | Y | Y | N | Y | Y | U | U | Y | NA | Moderate |
| Sharif 1991 | N | Y | N | Y | Y | U | U | Y | NA | Moderate |
| Ohara 1991 | Y | U | Y | Y | Y | Y | Y | Y | NA | High |
| Street 1991 | N | Y | N | N | Y | Y | Y | Y | NA | Moderate |
| Ozeren 1998 | N | N | N | N | Y | Y | U | Y | NA | Low |
| Holgado 1987 | N | N | N | Y | N | Y | U | Y | NA | Low |
| Liberthson 1986 | N | Y | N | N | Y | Y | Y | Y | NA | Moderate |
| Kumaki 1985 | Y | N | Y | Y | Y | N | U | Y | NA | Moderate |
| Warth 1985 | N | Y | N | N | Y | Y | U | Y | NA | Moderate |
| Beardsley 1982 | N | N | N | N | Y | Y | Y | Y | NA | Low |

Y: Yes; N: No; U: Unclear; NA: Not Applicable

Q1: Was the sample frame appropriate to address the target population

Q2: Were study participants sampled in an appropriate way

Q3: Was the sample size adequate

Q4: Were the study subjects and the setting described in detail

Q5: Was the data analysis conducted with sufficient coverage of the identified sample

Q6: Were valid methods used for the identification of the condition

Q7: Was the condition measured in a standard, reliable way for all participants

Q8: Was there appropriate statistical analysis

Q9: Was the response rate adequate, and if not, was the low response rate managed appropriately

Table 9: Risk of bias assessment summary table for syndromic prevalence studies

| Study | Q1 | Q2 | Q3 | Q4 | Q5 | Q6 | Q7 | Q8 | Q9 | Overall |
| --- | --- | --- | --- | --- | --- | --- | --- | --- | --- | --- |
| Backer 2006 | Y | U | N | Y | Y | Y | Y | Y | U | Moderate |
| Espinola-Zavaleta 2010 | N | Y | N | Y | Y | Y | U | Y | U | Moderate |
| Geva 1987 | N | Y | N | Y | Y | U | U | Y | NA | Moderate |
| Gu 2015 | N | Y | N | Y | Y | Y | Y | Y | NA | Moderate |
| Hirata 1992 | Y | U | N | Y | Y | Y | U | Y | NA | Moderate |
| Karnebeek 2001 | Y | Y | N | Y | Y | Y | U | NA? | NA | Moderate |
| Kunkala 2013 | N | Y | N | Y | Y | Y | Y | Y | NA | Moderate |
| Lopez 2015 | N | N | N | Y | Y | Y | Y | Y | U | Moderate |
| Mielczarek 2018 | Y | Y | N | Y | Y | N | U | Y | NA | Moderate |
| Mueller 2013 | N | Y | N | Y | Y | N | Y | U | U | Moderate |
| Muhlstadt 2019 | Y | Y | N | Y | Y | Y | U | Y | NA | Moderate |
| Ozdemir 2011 | N | N | N | Y | Y | Y | U | Y | N | Moderate |
| Rybczynski 2010 | Y | Y | Y | Y | Y | Y | Y | Y | Y | High |
| Taub 2009 | Y | Y | N | Y | Y | Y | Y | Y | NA | High |
| Yetman 2003 | Y | Y | N | Y | Y | N | U | Y | U | Moderate |
| Asher 2017 | Y | Y | Y | Y | Y | U | U | Y | NA | Moderate |
| Atzinger 2011 | Y | Y | Y | N | Y | U | U | Y | NA | Moderate |
| Dolan 1997 | Y | N | N | Y | Y | Y | U | Y | U | Moderate |
| Foltz 2019 | Y | Y | Y | Y | Y | U | N | Y | NA | Moderate |
| Forrest 1980 | Y | N | Y | Y | Y | N | U | Y | NA | Moderate |
| Piteri Toro 2023 | Y | Y | N | Y | Y | U | U | Y | NA | Moderate |
| Rashed 2022 | Y | Y | Y | Y | Y | U | U | Y | NA | Moderate |
| Shiari 2012 | N | Y | N | Y | Y | Y | U | Y | U | Moderate |
| Bruno 2023 | N | Y | N | Y | Y | Y | U | Y | U | Moderate |
| Cha 2019 | N | Y | N | Y | Y | Y | U | Y | NA | Moderate |
| Collins 2010 | Y | Y | Y | Y | Y | U | U | Y | NA | Moderate |
| Ergul 2012 | Y | Y | N | Y | Y | Y | U | Y | NA | Moderate |
| Lee 2022 | Y | Y | N | Y | Y | U | U | Y | NA | Moderate |
| Pasqua 2009 | Y | Y | N | Y | Y | Y | U | Y | Y | High |
| Scheiber 2006 | Y | N | N | Y | Y | Y | Y | Y | NA | Moderate |
| Smith 1988 | Y | N | N | Y | Y | N | U | Y | N | Moderate |


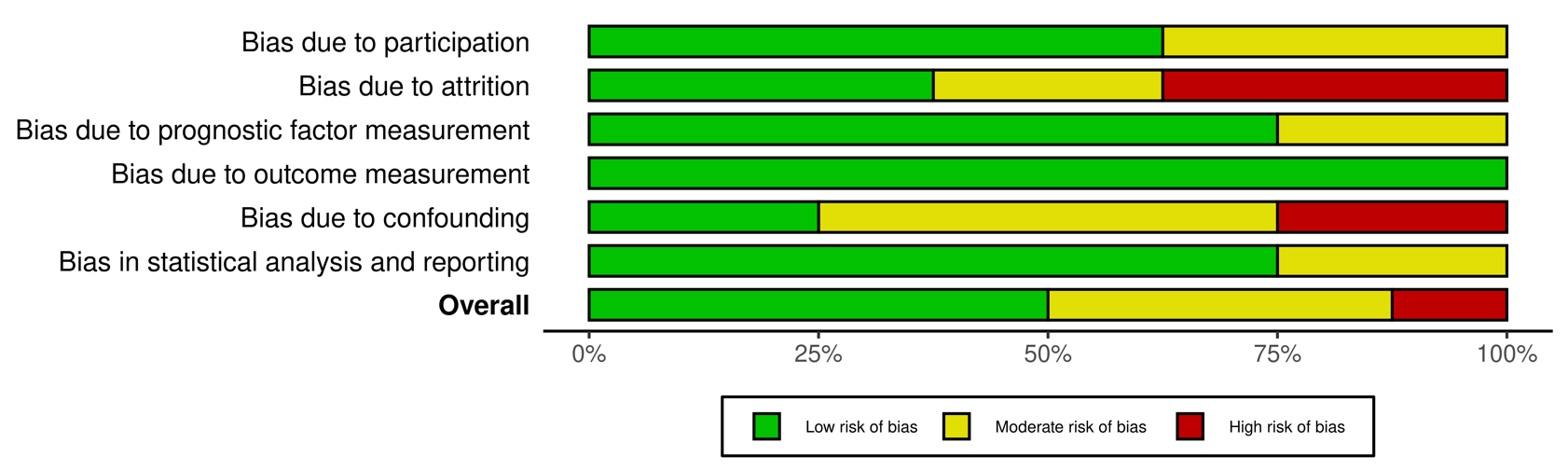


Figure 20: Risk of bias assessment graph for progression and outcome studies

**Bibliography**

1. Parvar SY, Ghaderpanah R, Naghshzan A. Prevalence of congenital heart disease according to the echocardiography findings in 8145 neonates, multicenter study in southern Iran. *Health Sci Rep* 2023;6:e1178

2. Dias G, Hafe P Von, Cardoso F, Pereira T, Tinoco M, Português J, et al. Usefulness of echocardiography in athletes: experience of a Portuguese center. *Cardiovascular and Metabolic Science* 2022;33:64–68.

3. Savis A, Simpson JM, Kabir S, Peacock K, Beardsley H, Sinha MD. Prevalence of cardiac valvar abnormalities in children and young people with autosomal dominant polycystic kidney disease. *Pediatric Nephrology* 2023;38:705–709.

4. Pfeferman MB, Rocha DR da, Rodrigues FG, Pfeferman E, Heilberg IP. Echocardiographic Abnormalities in Autosomal Dominant Polycystic Kidney Disease (ADPKD) Patients. *J Clin Med* 2022;11.

5. Fu CM, Wang JK, Wu MH, Hua YC, Chiu SN, Lin MT, et al. Changing spectrum of cardiac diseases in children: An extended longitudinal observation study of a pediatric cardiac screening program. *Acta Cardiol Sin* 2021;37:420–426.

6. Çağlayan U, Ramoğlu MG, Atalay S, Uçar T, Tutar E. Echocardiographic screening for mitral valve prolapse in Turkish school children. *Int J Cardiovas Imaging* 2021;37:1649–1657.

7. Liu PY, Tsai KZ, Lin YP, Lin CS, Zeng HC, Takimoto E, et al. Prevalence and characteristics of mitral valve prolapse in military young adults in Taiwan of the CHIEF Heart Study. *Sci Rep* 2021;11.

8. Hadi Alsaady AA, Nashtar SB, Hashim TM. Clinical study of mitral valve prolapse in Karbala Governorate, Iraq. *J Cardiovasc Dis Res* 2021;12:60–67.

9. Modaff DS, Hegde SM, Wyman RA, Rahko PS. Usefulness of Focused Screening Echocardiography for Collegiate Athletes. Am J Cardiol 2019;123:169–174.

10. Lang C, Wang R, Chen Z, He S, Zou Q, Wu J, et al. Incidence and Risk Factors of Cardiac Abnormalities in Patients with Idiopathic Scoliosis. *World Neurosurg* 2019;125:e824–e828.

11. Ong G, Connelly KA, Goodman J, Leong-Poi H, Evangelista V, Levitt K, et al. Echocardiographic Assessment of Young Male Draft-Eligible Elite Hockey Players Invited to the Medical and Fitness Combine by the National Hockey League. *Am J Cardiol* 2017;119:2088–2092.

12. Azami M, Yektakooshali MH, Reza M, Ahmadi H. Heart Valves Prolapse in Population Referred to Heart Clinic in Ilam, West of Iran. *Crescent Journal of biological and medical sciences* 2017;4

13. Bozcali E, Ucpunar H, Sevencan A, Balioglu MB, Albayrak A, Polat V. A retrospective study of congenital cardiac abnormality associated with scoliosis. *Asian Spine J* 2016;10:226–230.

14. Kang G, Xiao J, Wang Y, Wang J, Chen Y, Liu Q, et al. Prevalence and clinical significance of cardiac murmurs in schoolchildren. *Arch Dis Child* 2015;100:1028–1031.

15. Turker Y, Baltaci D, Basar C, Akkaya M, Ozhan H. The prevalence and clinical characteristics of mitral valve prolapse in a large population-based epidemiologic study: the MELEN study. *Eur Rev Med Pharmacol Sci*. 2015;19:2208-12.

16. Nishio S, Kusunose K, Yamada H, Yamao M, Hirata Y, Mori K, et al. Echocardiographic screening for congenital heart disease in 8819 children: A report from local community events for children’s healthcare. *J Cardio*l 2015;66:315–319.

17. Delling FN, Rong J, Larson MG, Lehman B, Osypiuk E, Stantchev P, et al. Familial clustering of mitral valve prolapse in the community. *Circulation* 2015;131:263–268.

18. Güvenç TS, Canga Y, Karabağ Y, Ozen K, Balcı B. Prevalence of mitral valve prolapse in residents living at moderately high altitude. *Wilderness Environ Med.* 2012;23:300-6.

19. Rizzo M, Spataro A, Cecchetelli C, Quaranta F, Livrieri S, Sperandii F, et al. Structural cardiac disease diagnosed by echocardiography in asymptomatic young male soccer players: Implications for pre-participation screening. *Br J Sports Med* 2012;46:371–373.

20. Tagarakis GI, Karantzis I, Tsolaki F, Stylianakis GE, Daskalopoulos ME, Tsilimingas NB. Classic and non-classic forms of mitral valve prolapse. *Anadolu Kardiyoloji Dergisi* 2012;12:2–4.

21. Ishikawa T, Iwashima S, Ohishi A, Nakagawa Y, Ohzeki T. Prevalence of congenital heart disease assessed by echocardiography in 2067 consecutive newborns*. Acta Paediatr.* 201;100:55-60.

22. Liu YT, Guo LL, Tian Z, Zhu WL, Yu B, Zhang SY, et al. A retrospective study of congenital scoliosis and associated cardiac and intraspinal abnormities in a Chinese population. *European Spine Journal* 2011;20:2111–2114.

23. Sattur S, Bates S, Movahed MR. Prevalence of mitral valve prolapse and associated valvular regurgitations in healthy teenagers undergoing screening echocardiography. *Exp Clin Cardiol*. 2010;15:13-5.

24. Hepner AD, Morrell H, Greaves S, Greaves J, Movahed MR. Prevalence of mitral valvar prolapse in young athletes. *Cardiol Young* 2008;18:402–404.

25. Strader JR, Harrell TW, Adair A, Kruyer WB. Efficacy of echocardiographic screening of pilot applicants. *Aviat Space Environ Med* 2008;79:514–517.

26. Hepner AD, Ahmadi-Kashani M, Movahed MR. The prevalence of mitral valve prolapse in patients undergoing echocardiography for clinical reason. *Int J Cardiol* 2007;123:55–57.

27. Bar-Dayan Y, Elishkevits K, Goldstein L, Goldberg A, Ohana N, Onn E, et al. The prevalence of common cardiovascular diseases among 17-year-old Israeli conscripts. *Cardiology* 2005;104:6–9.

28. Devereux RB, Jones EC, Roman MJ, Howard BV, Fabsitz RR, Liu JE, et al. Prevalence and correlates of mitral valve prolapse in a population-based sample of American Indians: the Strong Heart Study. *Am J Med* 2001;111:679-85.

29. Balderas FJ, Rubi G, Hinojosa P, Arellano, Yanez P, Sanchez ML, et al. Two-Dimensional Echo Doppler Findings in Juvenile and Adult Onset Ankylosing Spondylitis with Long-Term Disease. *Angiology* 2001;52:543-548

30. Flack JM, Kvasnicka JH, Gardin JM, Gidding SS, Manolio TA, Jacobs DR Jr. Anthropometric and physiologic correlates of mitral valve prolapse in a biethnic cohort of young adults: the CARDIA study. *Am Heart J*. 1999;138:486-92.

31. Freed LA, Levy D, Levine RA, Larson MG, Evans JC, Fuller DL, et al. Prevalence and Clinical Outcome of Mitral-Valve Prolapse*. N Eng J Med* 1999; 134:1-7.

32. Hossack KF, Leddy CL, Johnson AM, Schrier RW, Gabow P. Echocardiographic findings in autosomal dominant polycystic kidney disease. *N Eng J Med* 1988; 319:907-912

33. Dhuper S, Ehlers KH, Fatica NS, Myridakis DJ, Klein AA, Friedman DM, et al. Incidence and risk factors for mitral valve prolapse in severe adolescent idiopathic scoliosis*. Pediatr Cardiol*. 1997;18:425-8.

34. Nascimento R, Freitas A, Teixeira F, Pereira D, Cardoso A, Dinis M, Mendonça I. Is mitral valve prolapse a congenital or acquired disease. *J Am Coll Cardiol* 1997; 79:226-227.

35. Kahaly G, Mohr-Kahaly S, Beyer J, Meyer J. Prevalence of myxomatous mitral valve prolapse in patients with lymphocytic thyroiditis. Am J Cardiol. 1995;76:1309-10.

36. Ivy DD, Shaffer EM, Johnson AM, Kimberling WJ, Dobin A, Gabow PA. Cardiovascular Abnormalities in Children with Autosomal Dominant Polycystic Kidney Disease. *J Am Soc Nephrol* 1995; 5:2032-2036.

37. Gupta R, Jain BK, Gupta HP, Ranawat SS, Sharma AK, Gupta KD. Mitral Valve Prolapse: Two dimensional echocardiography reveals a high prevalence in three two twelve year old children. *Indian Pediatrics* 1991; 29:417-422

38. Sharif KW, Casey TA, Coltart J. Prevalence of mitral valve prolapse in keratoconus patients. *J R Soc Med*. 1992;85:446-8.

39. Ohara N, Mikajima T, Takagi J, Kato H. Mitral valve prolapse in childhood: the incidence and clinical presentations in different age groups. *Acta Paediatr Jpn*. 1991;33:467-75.

40. Street DA, Vinokur ET, Waring GO, Pollak SJ, Clements SD, Perkins J V. Lack of Association Between Keratoconus, Mitral Valve Prolapse, and Joint Hypermobility. *Ophthalmology* 1991;98:170–176.

41. Ozeren A, Türkoğlu C, Saygili R. Low prevalence of mitral valve prolapse in bipolar affective disorder. *Acta Psychiatr Scand*. 1986;74:605-6.

42. Holgado GM, Prakash R. Prevalence of mitral valve prolapse in hypertension. J Natl Med Assoc. 1987;79:966-8.

43. Liberthson R, Sheehan DV, King ME, Weyman AE. The prevalence of mitral valve prolapse in patients with panic disorders. Am J Psychiatry. 1986;143:511-5.

44. De Backer J, Loeys B, Devos D, Dietz H, De Sutter J, De Paepe A. A critical analysis of minor cardiovascular criteria in the diagnostic evaluation of patients with Marfan syndrome. *Genet Med*. 2006;8:401-8.

45. Espínola-Zavaleta N, Iqbal FM, Nanda NC, Enríquez-Rodríguez E, Amezcua-Guerra LM, Bojalil-Parra R, Reyes PA, Soto ME. Echocardiographic study of a Mestizo-Mexican population with Marfan syndrome. *Echocardiography.* 2010;27:923-30.

46. Geva T, Hegesh J, Frand M. The clinical course and echocardiographic features of Marfan's syndrome in childhood. *Am J Dis Child.* 1987;141:1179-82.

47. Gu X, He Y, Li Z, Han J, Chen J, Nixon JV. Echocardiographic versus histologic findings in Marfan syndrome. *Tex Heart Inst J*. 2015;42:30-4.

48. Hirata K, Triposkiadis F, Sparks E, Bowen J, Boudoulas H, Wooley CF. The Marfan syndrome: cardiovascular physical findings and diagnostic correlates. *Am Heart J*. 1992;123:743-52.

49. Van Karnebeek CD, Naeff MS, Mulder BJ, Hennekam RC, Offringa M. Natural history of cardiovascular manifestations in Marfan syndrome. Arch Dis Child. 2001;84:129-37.

50. Kunkala MR, Schaff HV, Li Z, Volguina I, Dietz HC, LeMaire SA, et al. Mitral valve disease in patients with Marfan syndrome undergoing aortic root replacement. *Circulation*. 2013;128:S243-7.

51. Lopez VM, Perez AB, Moisés VA, Gomes L, Pedreira Pda S, Silva CC, et al. Avaliação clínico-cardiológica e ecocardiográfica, seqüencial, em crianças portadoras da síndrome de Marfan [Serial clinical and echocardiographic evaluation in children with Marfan syndrome]. *Arq Bras Cardiol*. 2005;85:314-8.

52. Wozniak-Mielczarek L, Sabiniewicz R, Drezek-Nojowicz M, Nowak R, Gilis-Malinowska N, Mielczarek M, Łabuc A, Waldoch A, Wierzba J. Differences in Cardiovascular Manifestation of Marfan Syndrome Between Children and Adults. *Pediatr Cardiol* 2019;40:393–403.

53. Mueller GC, Stark V, Steiner K, Kodolitsch Y Von, Rybczynski M, Weil J, Mir TS. Impact of age and gender on cardiac pathology in children and adolescents with marfan syndrome. *Pediatr Cardiol* 2013;34:991–998.

54. Mühlstädt K, Backer J De, Kodolitsch Y von, Kutsche K, Mosquera LM, Brickwedel J, et al. Case-matched comparison of cardiovascular outcome in loeys-dietz syndrome versus Marfan syndrome. *J Clin Med* 2019;8.

55. Ozdemir O, Olgunturk R, Kula S, Tunaoglu FS. Echocardiographic findings in children with Marfan syndrome. Cardiovasc J Afr 2011;22:245–248.

56. Rybczynski M, Mir TS, Sheikhzadeh S, Bernhardt AMJ, Schad C, Treede H, et al. Frequency and age-related course of mitral valve dysfunction in the marfan syndrome. *Am J of Cardiol* 2010;106:1048–1053.

57. Taub CC, Stoler JM, Perez-Sanz T, Chu J, Isselbacher EM, Picard MH, et al. Mitral valve prolapse in marfan syndrome: An old topic revisited. *Echocardiography* 2009;26:357–364.

58. Yetman AT, Bornemeier RA, McCrindle BW. Long-term outcome in patients with Marfan syndrome: is aortic dissection the only cause of sudden death? J Am Coll Cardiol. 2003;41:329-32.

59. Asher SB, Chen R, Kallish S. Mitral valve prolapse and aortic root dilation in adults with hypermobile Ehlers–Danlos syndrome and related disorders. *Am J Med Genet* 2018;176:1838–1844.

60. Atzinger CL, Meyer RA, Khoury PR, Gao Z, Tinkle BT. Cross-sectional and longitudinal assessment of aortic root dilation and valvular anomalies in hypermobile and classic Ehlers-Danlos syndrome. *J Pediatr*. 2011;158:826-830

61. Dolan AL, Mishra MB, Chambers JB, Grahame R. Clinical and echocardiographic survey of the Ehlers-Danlos syndrome. Br J Rheumatol 1997;36:459–462.

62. Rauser-Foltz KK, Starr LJ, Yetman AT. Utilization of echocardiography in Ehlers-Danlos syndrome. *Congenit Heart Dis* 2019;14:864–867.

63. Jessee EF, Owen DS Jr, Sagar KB. The benign hypermobile joint syndrome. *Arthritis Rheum*. 1980;23:1053-6.

64. Pietri-Toro JM, Gardner OK, Leuchter JD, DiBartolomeo G, Hunter JA, Forghani I. Prevalence of cardiovascular manifestations in patients with hypermobile Ehlers-Danlos syndrome at the University of Miami. *Am J Med Genet* A 2023;191:1502–1507.

65. Rashed ER, Ruiz Maya T, Black J, Fettig V, Kadian-Dodov D, Olin JW, et al. Cardiovascular manifestations of hypermobile Ehlers–Danlos syndrome and hypermobility spectrum disorders. *Vascular Medicine* 2022;27:283–289.

66. Bruno E, Rossi N, Thüer O, Córdoba R, Alday LE. Cardiovascular findings, and clinical course, in patients with Williams syndrome. *Cardiol Young* 2003;13:532–536.

67. Cha SG, Song MK, Lee SY, Kim GB, Kwak JG, Kim WH, et al. Long-term cardiovascular outcome of Williams syndrome. *Congenit Heart Dis* 2019;14:684–690.

68. Collins RT 2nd, Kaplan P, Somes GW, Rome JJ. Long-term outcomes of patients with cardiovascular abnormalities and williams syndrome. *Am J Cardiol*. 2010;105:874-8.

69. Ergul Y, Nisli K, Kayserili H, Karaman B, Basaran S, Koca B, et al. Cardiovascular abnormalities in Williams syndrome: 20 years’ experience in Istanbul. *Acta Cardiol* 2012;67:649–655.

70. Lee CL, Lin SM, Chen MR, Chuang CK, Syu YM, Chiu HC, et al. Long-Term Cardiovascular Findings in Williams Syndrome: A Single Medical Center Experience in Taiwan. *J Pers Med*. 2022;12:817.

71. Pasqua A Del, Rinelli G, Toscano A, Iacobelli R, Digilio C, Marino B, et al. New findings concerning cardiovascular manifestations emerging from long-term follow-up of 150 patients with the Williams-Beuren-Beuren syndrome. *Cardiol Young* 2009;19:563–567.

72. Scheiber D, Fekete G, Urban Z, Tarjan I, Balaton G, Kosa L, et al. Echocardiographic findings in patients with Williams-Beuren syndrome. Wien Klin Wochenschr 2006;118:538–542.

73. Smith KA, Karas S. Cardiac anomalies in Williams-Beuren syndrome. Arch Dis Child. 1988;63:809-13.

74. Ma JI, Igata S, Strachan M, Nishimura M, Wong DJ, Raisinghani A, et al. Predictive Factors for Progression of Mitral Regurgitation in Asymptomatic Patients With Mitral Valve Prolapse. *Am J Cardiol*. 2019;123:1309-1313.

75. Delling FN, Rong J, Larson MG, Lehman B, Fuller D, Osypiuk E, et al. Evolution of Mitral Valve Prolapse: Insights From the Framingham Heart Study. *Circulation* 2016;133:1688–1695.

76. Avierinos JF, Detaint D, Messika-Zeitoun D, Mohty D, Enriquez-Sarano M. Risk, determinants, and outcome implications of progression of mitral regurgitation after diagnosis of mitral valve prolapse in a single community. *Am J Cardiol*. 2008;101:662-7.

77. Wand O, Prokupetz A, Grossman A, Assa A. Natural history of mitral valve prolapse in military aircrew. *Cardiology*. 2011;118:50-4.

78. Kamei F, Nakahara N, Yuda S, Kobayashi N, Tsuchihashi K, Shimamoto K. Long-term site-related differences in the progression and regression of the idiopathic mitral valve prolapse syndrome. *Cardiology*. 1999;91:161-8.

79. Kim S, Kuroda T, Nishinaga M, Yamasawa M, Watanabe S, Mitsuhashi T, et al. Relationship between severity of mitral regurgitation and prognosis of mitral valve prolapse: echocardiographic follow-up study. *Am Heart J*. 1996;132:348-55.

80. Murakami H, Yonekura S, Sudoh K, Hikita N, Nagao K, Takahashi N, et al. Site-Related Difference in the Prevalence of Mitral Valve Prolapse. Relation to the Prevalence and Severity of Mitral Regurgitation. *Jpn Heart J* 1991; 32:785-798.

81. Avierinos JF, Gersh BJ, Melton LJ 3rd, Bailey KR, Shub C, Nishimura RA, et al. Natural history of asymptomatic mitral valve prolapse in the community. *Circulation.* 2002;106:1355-61.
